# Exact box-counting and temporal sampling algorithms for fractal dimension estimation with applications to animal behavior analysis

**DOI:** 10.1016/j.rineng.2024.103755

**Published:** 2024-12-19

**Authors:** Tao Cui, Tingting Wang

**Affiliations:** Department of Pharmacology and Physiology, Georgetown University Medical Center, Washington, 20007, D.C., USA

**Keywords:** Fractal dimension, Box-counting, Movement complexity, Psychiatric disorder, Schizophrenia, Dysbindin, Homeostatic plasticity

## Abstract

Fractal dimension (FD) is a widely recognized metric in mathematics and physics for quantifying the complexity of intricate objects and processes. In this study, we propose novel algorithms to estimate the FD of animal movement using high-resolution spatial and temporal data. To enhance estimation accuracy, we developed an oversampling technique that linearly interpolates between adjacent points on movement paths. Furthermore, we introduced an exact box-counting algorithm tailored for piecewise linear paths, ensuring accurate fractal dimension estimations. Considering that animal behavior is typically recorded at fixed frame rates, we also propose a temporal sampling method for calculating FD using temporal domain scales. Recognizing that FD varies with scale, we employ a dual total least squares method to identify the optimal scale for FD comparisons across different genotypes. Through large-scale experiments on the movement of *Drosophila melanogaster* larvae, we show that mutations in the schizophrenia-associated gene *Dysbindin* significantly increase FD, suggesting potential impairments in motor function. These findings highlight FD as a robust and quantitative metric for assessing the complexity of movement behavior.

## 1. Introduction

Understanding how genetic information encodes animal behaviors remains a fundamental question in biology. Recent advances in autotracking technologies have greatly improved the efficiency of behavioral data collection [1], providing valuable insights into the mechanisms through which various genes influence animal behavior. These developments are crucial for unraveling the complexities of brain function and the molecular underpinnings of a wide range of neurological and neuropsychiatric disorders. Despite these advancements, a precise mathematical framework to describe the intricate movement patterns of animals is still lacking. The concept of fractal dimension (FD), extensively utilized in fields such as mathematics [2], physics [3–5], coastal geography [6,7], biopolymeric products [8], and engineering [9,10], provides a powerful tool for characterizing the complexity, scaling properties, and self-similarity of geometric forms. Recently, a new fuzzy fractal dimension was introduced in [11]. FD has also proven valuable in the analysis of chaotic dynamical systems and stochastic processes [2–5]. Despite these advancements, the application of FD to understanding the complexity of animal movement behavior remains in its early stages.

FD is a robust metric for assessing path tortuosity, independent of an animal’s shape metrics, such as bend angle and length, which are prone to noise and challenging to measure precisely. Moreover, animal movements are often captured in high-frame-rate videos, and FD represents the minimal set of independent variables required to model these time series, facilitating its quantification in the temporal domain. Although FD has been applied in various biological fields, its use in addressing biological questions is not without limitations [12–14]. A significant constraint is the inability to sample biological objects at infinite time scales, which severely impacts the effectiveness of commonly used methods, such as box-counting and correlation sum [3]. Additionally, estimating FD from pixelated images is limited by finite scale size and is prone to significant quantization errors due to rounding all points to integer pixel indices [9,15]. Furthermore, the placement of box grids can substantially influence FD quantification, yet this issue has not been thoroughly investigated. While numerous biological studies require comparisons of behavioral phenotypes across various genotypes or conditions, the use of FD to quantify behavioral phenotypes associated with different genotypes remains minimally explored.

Muscle contractions and motor movements in *Drosophila* larvae are orchestrated by neuromuscular junctions (NMJs), which function as glutamatergic synapses [16,17]. A key component of these synapses, *GluRIIA*, is a subunit of glutamate receptors located at the NMJ, playing a critical role in muscle contraction and motor activities. Disruptions in the functionality of these glutamate receptors, caused by pharmacological or genetic alterations, trigger an increase in presynaptic neurotransmitter release. This compensatory mechanism, designed to counterbalance changes in postsynaptic glutamate receptor activity, helps maintain postsynaptic excitability and, consequently, motor functions [18–20]. This adaptive response is termed Presynaptic Homeostatic Potentiation (PHP).

Dystrobrevin-binding protein (Dysbindin) is a component of the Dystrophin-associated Protein Complex (DPC) in skeletal muscle cells and is also part of the Biogenesis of Lysosome-related Organelles Complex 1 (BLOC-1) [21–23]. *Dysbindin* is essential for regulating intracellular trafficking, and its deficiency affects synaptic transmission and plasticity, with strong associations to schizophrenia [24–27]. *Ada2b*, a critical component of the Spt-Ada-Gcn5 acetyltransferase protein complex, is required for histone acetylation [28,29]. Notably, both *Dysb* and *Ada2b* play pivotal roles in regulating PHP at the NMJ [20,30]. In this study, we investigate how *GluRIIA, Dysb*, and *Ada2b* affect movement behavior in *Drosophila* using FD.

We develop a novel oversampling algorithm to address the issue of finite sample numbers in the image pixelation method. Additionally, by representing the time series of animal movement as a piecewise linear curve, we propose a new exact box-counting algorithm capable of determining the precise number of boxes intersecting the movement curve. Since our data are derived from videos recorded at a constant high frame rate, resulting in uniformly sampled animal paths over time, we introduce a temporal sampling algorithm to estimate FD by downsampling the time series at various time intervals. We comprehensively evaluate the performance of existing methods and proposed algorithms in estimating FD using large-scale data, consisting of 230 movement trajectories of *Drosophila* larvae, including both *wild-type* and mutant strains such as *GluRIIA, Dysb*, and *Ada2b*.

Our analysis reveals the limitations of existing algorithms and rigorously examines the impact of varying box grid origins and rotation angles through perturbation analysis. The results reveal that the original box-counting algorithm tends to underestimate the FD with greater variability, potentially masking FD differences across genotypes. In contrast, our proposed exact box-counting and temporal sampling algorithms demonstrate effectiveness in quantifying FD differences between genotypes. Notably, *Dysbindin* mutants consistently exhibit higher FD values and more complex movement trajectories compared to the *wild-type* control. In summary, we have developed new mathematical methods and scalable computational algorithms for precisely quantifying biological complexity using FD. This metric provides a valuable tool for investigating animal behavior across different genotypes. Our results confirm the utility of fractal dimension in quantifying the genetic underpinnings of movement complexity, potentially revealing mechanisms implicated in motor dysfunction across neurological and psychiatric disorders. The proposed algorithms can be easily adapted and readily used in other species and other areas of biology, mathematics, physics, and other engineering fields. This work paves the way for broader applications of fractal dimension in biomedical research, particularly in elucidating complex brain functions and their molecular etiologies.

## 2. Materials and methods

### 2.1. Definitions of FD and the original box-counting algorithm

#### The Hausdorff dimension:

Let X be any non-empty subset of the *d*-dimensional Euclidean space $R^{d}$, i.e. $X\subset R^{d}$. The diameter of X is defined as the greatest distance between any two points in X, i.e., $|X|=sup\{|x-y|:x,y\in X\}.\;\left\{B_{i}\right\}$ is an ϵ-cover of X if $\left\{B_{i}\right\}$ is a countable (or finite) collection of sets of diameter at most ϵ that cover X, i.e. $X\subset \cup_{i}B_{i}$ with $\left|B_{i}\right|\leq \epsilon ,\forall i$. For any δ>0,

(1) $$H^{\delta }(X)=\underset{\epsilon \to 0}{\lim}\inf\left\{\overset{\infty }{\underset{i=0}{\sum }}{\left|B_{i}\right|}^{\delta }:\left\{B_{i}\right\}\;\text{is an}\;\epsilon \;\text{cover of}\;X\right\}.$$

The Hausdorff dimension $D_{H}$ of $X\subset R^{d}$ is a critical value of δ at which $H^{\delta }(X)$ jumps from ∞ to 0, i.e. $H^{\delta }(X)=\infty$ if $\delta <D_{H}$ and $H^{\delta }(X)=0$ if $\delta >D_{H}$ [2].

#### The box-counting dimension:

A special covering is a fixed-size grid consisting of cubes of width ϵ in $R^{d}$, i.e. cubes of the form

(2) $$\left[m_{1}\epsilon ,\left(m_{1}+1\right)\epsilon \right)\times \cdots \times \left[m_{d}\epsilon ,\left(m_{d}+1\right)\epsilon \right),$$

where $m_{1},\ldots ,m_{d}$ are integers. Let N(ϵ) be the number of cubes that intersect X. The box-counting dimension is

(3) $$D_{B}=\underset{\epsilon \to 0}{\lim}\frac{\text{log}N(\epsilon )}{-\text{log}\epsilon }.$$

As the box covering is a special covering, it is shown in [31, (3.17)] that

(4) $$D_{H}\leq D_{B},$$

Given that Hausdorff and box-counting dimensions are equivalent for most sets of reasonable regularity that interest us, we refer to both dimensions collectively as the FD, symbolized by D.

A natural and direct approximation to compute $D_{B}$ is to apply (3) directly using the smallest ϵ available. Let

(5) $$N(\epsilon )∼C\epsilon^{-D},$$

we have

(6) $${\hat{D}}_{B}\approx \frac{\text{log}N(\epsilon )}{-\text{log}\epsilon }=D-\frac{\text{log}C}{\text{log}\epsilon }.$$

However, the expression $\frac{\log C}{\log\epsilon }$ decreases logarithmically slowly as ϵ approaches zero. A better and widely adopted method is to write (5) as

(7) logN(ϵ)=−Dlogϵ+logC.

After calculating N(ϵ) for a suitable range of ϵ, we run a linear regression of log⁡N(ϵ) over log⁡ϵ and estimate D as the negative of the slope.

We consider biological processes, such as animal movement paths in a time series, represented as $f(t)=\left(f_{1}(t),\ldots ,f_{d}(t)\right)\in R^{d}$. A set of n points $x_{i}=\left(x_{i,1},\ldots ,x_{i,d}\right)\in R^{d},i=1,\ldots ,n$ on f(t) is observed. When uniform sampling in time is used (constant frame rate videos), we have $x_{i}=f(i\Delta t)\left(x_{i,j}=f_{j}(i\Delta t),j=1,\ldots ,d\right)$, where Δt is the sampling interval.

In (5), a homogeneous FD is assumed to be independent of ϵ. However, we find that FD typically depends on the scale ϵ, which we denote as D(ϵ). Unlike in mathematical and physical applications, where the focus is often on the limit as ϵ→0 in (3), biological systems are constrained by the resolution of measurement devices. Additionally, action potentials generally fire at a maximum rate of a few hundred Hertz, and neuronal circuits may operate at even lower frequencies, suggesting that extremely small temporal scales might lack biological relevance. Thus, we are particularly interested in the local FD at specific scales. This raises critical questions: at what scale should the FD be computed, and how can different animal movement traces be aligned to ensure their FDs are comparable? These questions are addressed in Section 3.3.

#### The original box-counting algorithm:

The core of box-counting algorithm is to compute N(ϵ). For each $x_{i}$, we find its cube (box) index $m_{i}\in Z^{d}$ as

(8) $$m_{i}=\left(m_{i,1},\ldots ,m_{i,d}\right)=\left(\left\lfloor \frac{x_{i,1}}{\epsilon }\right\rfloor ,\ldots ,\left\lfloor \frac{x_{i,d}}{\epsilon }\right\rfloor \right),$$

where

(9) $$m_{i,j}=\left\lfloor \frac{x_{i,j}}{\epsilon }\right\rfloor$$

and ⌊⋅⌋ is the floor function. N(ϵ) is then the number of distinct $m_{i}$. (8) corresponds to the cube in (2). The box-counting dimension $D_{B}$ can be determined by computing N(ϵ) for a suitable set of scales $\left\{\epsilon_{k}:k=\right.1,\ldots ,K\}$ and obtaining the negative slope of the log⁡N(ϵ) versus log⁡ϵ plot using linear regression. To implement the box-counting algorithm, we utilize a hash table data structure to store $m_{i}$, achieving an average time complexity of O(n). The number of distinct boxes is represented as $C\epsilon^{-D}\leq n$, resulting in a space complexity of $O\left(\epsilon^{-D}\right)$, which improves efficiency compared to the original method described in [3].

### 2.2. Proposed FD algorithms

#### 2.2.1. Oversampling algorithm

To address the underestimation of N(ϵ) and $D_{B}$ by the original box-counting method, which results from the limited number of observed points along the animals’ movement paths, we have developed an oversampling algorithm that enhances the sampling of adjacent points on these paths. The method begins by linking each pair of consecutive points in $\left\{x_{i}\right\}$ with a straight line, approximating the entire curve as piecewise linear. We then evenly distribute S points along the line segment connecting each pair of neighboring points. Assuming that the animal moves at a constant speed between two consecutive points (video frames with minimal intervals), and particularly when the sampling interval Δt is minimal, each of the S intermediate points between $x_{i}$ and $x_{i+1}$ can be considered as representing points sampled at an interval of Δt/(S+1). This approach effectively increases the sampling rate by a factor of S+1.

To determine the S points, we parameterize the line using the normalized distance from the initial point. Formally, for each pair of points $\left(x_{i},x_{i+1}\right)$, the line between $\left(x_{i},x_{i+1}\right)$ is

(10) $$g_{i}(t)=x_{i}+t\left(x_{i+1}-x_{i}\right),t\in [0,1].$$

The S new points are $g_{i}(s/(S+1)),s=1,\ldots ,S$. Together with $\left\{x_{i}\right\}$, we get a new set of points of size (n-1)S+n as

(11) $$z_{i(S+1)+s}=g_{i}\left(\frac{s}{S+1}\right),i=1,\ldots ,n-1,s=0,\ldots ,S,\;\text{and}\;z_{n(S+1)}=x_{n}.$$

**Fig. 1.**
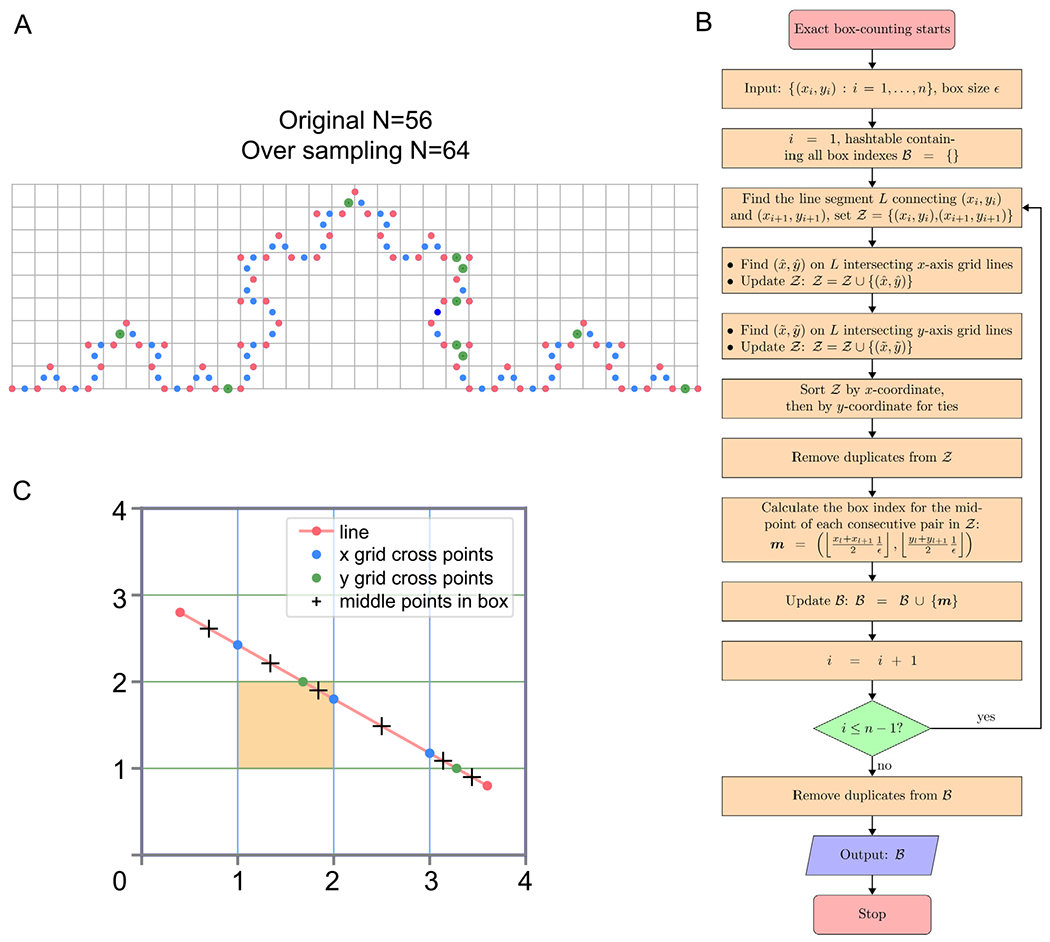
Illustration of proposed oversampling and exact box-counting methods. **A**. An example to show the oversampling of the Koch curve (level 3). Red dots represent points generated directly by the Koch curve (S=0), while blue and green dots are oversampled points (S=1). Note that green dots are located in boxes that do not contain any red dot. Using only the red dots results in a count of 56 boxes, whereas incorporating the oversampled points increases the count to 64 boxes. **B**. Flowchart showing the steps computing FD using the exact box-counting method. **C**. An example to show the exact box-counting method. A linear line segment of a piecewise linear curve (red line) between red points intersects with the blue grid on the *x*-dimension (blue dots) and with the green grid on the *y*-dimension (green dots). The middle points between two neighboring dots intersect with the *x*- and/or *y*-dimension are shown in black crosses. The floor function is used to identify corresponding box index in Z, while the direct use of box indices would fail to identify all boxes intersected by the line, such as the “orange box”. Note that each linear segment of a piecewise linear curve is monotonic in either x and/or y.

When S=0,z reduces to x. The original box counting method can be applied to this oversampled set of points z. When we apply box-counting to a level 3 Koch curve by oversampling (S=1), we get 64 boxes (blue and green dots containing boxes, Fig. 1A), while we only get 56 boxes using the original data points on the curve (red dots, Fig. 1A). In this case, where lines connect adjacent points, the blue and green dots are indeed situated on the Koch curve itself. Therefore, relying solely on the red points underestimates the number of boxes.

#### 2.2.2. Exact box-counting algorithm

We can use an infinite number of points along the line (10) to precisely calculate the total count of boxes intersecting the piecewise linear curve. Detecting whether a box intersects with the curve reduces to verifying the presence of at least one curve point within the box. Therefore, the fundamental task of the exact box-counting algorithm is to determine if a box contains at least one point on the curve. The existing Bresenham’s line algorithm [32] approximates a straight line between two points on a 2-dimensional square grid by computing the coordinates of the pixels that form the line segment. However, this algorithm does not always include all the pixels, or “boxes,” that intersect with the line (Fig. S1).

For any 2-dimensional case, the sampled points are denoted as $\left(x_{i},y_{i}\right)$, and ℬ represents the hashtable storing all box indices. For each line segment connecting consecutive points, $\left(x_{i},y_{i}\right)\to \left(x_{i+1},y_{i+1}\right)$, where i=1,…,n-1, we determine all crossing points on the box boundaries (equivalently, the intersection points on the grid lines). Formally, in the x-dimension, if $x_{i}\neq x_{i+1}$, we locate all crossing points l such that:

(12) $$x_{i}<l\epsilon <x_{i+1}.$$

Let ${\overset{ˆ}{x}}_{i}=l\epsilon$ for each l satisfying (12). We find the corresponding ${\overset{ˆ}{y}}_{l}$ on the line segment between $\left(x_{i},y_{i}\right)$ and $\left(x_{i+1},y_{i+1}\right)$ using

(13) $${\hat{y}}_{l}=\frac{y_{i+1}-y_{i}}{x_{i+1}-x_{i}}\left({\hat{x}}_{l}-x_{i}\right)+y_{i}.$$

Similarly, in the y-dimension, if $y_{i}\neq y_{i+1}$, we find all m such that:

(14) $$y_{i}<m\epsilon <y_{i+1}.$$

Let ${\tilde{y}}_{m}=m\epsilon$ for each m satisfying (14). We then compute the corresponding ${\tilde{x}}_{m}$ on the line segment between $\left(x_{i},y_{i}\right)$ and $\left(x_{i+1},y_{i+1}\right)$ using

(15) $${\tilde{x}}_{m}=\frac{x_{i+1}-x_{i}}{y_{i+1}-y_{i}}\left({\tilde{y}}_{m}-y_{i}\right)+x_{i}.$$

We combine all points $\left({\overset{ˆ}{x}}_{l},{\overset{ˆ}{y}}_{l}\right)$ and $\left({\tilde{x}}_{m},{\tilde{y}}_{m}\right)$ into a list $𝒵=\left[\left(x_{i},y_{i}\right)\right.,\left.\left(x_{i+1},y_{i+1}\right),\left({\overset{ˆ}{x}}_{l},{\overset{ˆ}{y}}_{l}\right),\cdots ,\left({\tilde{x}}_{m},{\tilde{y}}_{m}\right),\cdots \right]$. The list 𝒵 is then sorted in ascending order by the first dimension (x), and for points with identical *x*-coordinates, by the second dimension (y) to address cases where points lie on a vertical line. Duplicates, which occur when a line passes through the corner of a box (i.e., $\left({\overset{ˆ}{x}}_{l},{\overset{ˆ}{y}}_{l}\right)=\left({\tilde{x}}_{m},{\tilde{y}}_{m}\right)$ in $\left.𝒵\right)$, are removed. After deduplication, the box index for the midpoint of each consecutive pair in 𝒵 is determined using

(16) $$m_{l}=\left(\left\lfloor \frac{x_{l}+x_{l+1}}{2}\frac{1}{\epsilon }\right\rfloor ,\left\lfloor \frac{y_{l}+y_{l+1}}{2}\frac{1}{\epsilon }\right\rfloor \right),$$

where $\left(x_{l},y_{l}\right)\in 𝒵$ and $\left(x_{l+1},y_{l+1}\right)\in 𝒵$. The index $m_{l}$ from (16) is added to the set ℬ. This process is repeated for all line segments 1≤i≤n-1. The total count N(ϵ) is then calculated as the cardinality of ℬ. A flowchart illustrating the exact box-counting algorithm is provided in Fig. 1B.

Except for the endpoints, a line is monotonic in either x and/or y, and it must cross into and out of a box through two points of intersection. The consecutive points in 𝒵 represent the two locations where the line intersects the box’s boundary (excluding the first and last points). The midpoint between these intersection points lies both on the line and within the box, ensuring that a point is identified inside each box intersected by the line. It is important to note that the points in 𝒵 are not directly used to assign box indices, as this approach would fail to capture all the boxes intersected by the line. For example, certain boxes, such as the “orange box,” would be overlooked if only the points in 𝒵 were used (Fig. 1C).

#### 2.2.3. Temporal sampling algorithm

The divider method, one of the earliest approaches proposed for computing FD, uses rulers of varying lengths to measure the length of a plane curve [6,33]. However, applying this method to a piecewise linear curve poses challenges, particularly in accurately determining the points where the ruler intersects each line segment. Given that animal movement data are typically recorded at a constant frame rate, we investigate the potential of using temporal rulers with varying time intervals instead of traditional spatial rulers. Intuitively, one might attempt to measure the curve’s length using different time intervals and define the FD as the slope of the log curve length versus the log time interval plot. However, this approach fails to produce a valid FD, as defining a time-domain FD proves to be a complex and non-trivial task.

Let U(δ) denote the average distance between two points separated by δ time steps, corresponding to a time difference of δΔt. We compute U(δ) as:

(17) $$U(\delta )=\frac{1}{m(\delta )}\overset{m(\delta )}{\underset{i=1}{\sum }}\left\|x_{i\delta +1}-x_{(i-1)\delta +1}\right\|=\frac{L(\delta )}{m(\delta )},$$

where ‖⋅‖ is the 2-norm, and

(18) $$m(\delta )=\left\lfloor \frac{n-1}{\delta }\right\rfloor ,$$

and

(19) $$L\left(\delta \right)=\overset{m\left(\delta \right)}{\underset{i=1}{\sum }}\left\|x_{i\delta +1}-x_{\left(i-1\right)\delta +1}\right\|$$

is the total length of the curve when measured using every δ frames, which is equivalent to temporal downsampling of the curve by a factor of δ.

Assuming that U(δ) follows the scaling law:

(20) $$U(\delta )=C\delta^{\alpha },$$

the fractal dimension $D_{T}$ derived from temporal downsampling of the curve is:

(21) $$D_{T}=1+(1-\alpha )\text{min}\left(d,\frac{1}{\alpha }\right).$$

When d=2 and $\alpha >0.5,D_{T}$ simplifies to:

(22) $$D_{T}=\frac{1}{\alpha }.$$

To estimate α from (20), we compute U(δ) for a suitable set of values $\left\{\delta_{k}:k=1,\ldots ,K\right\}$ and determine the slope of log⁡U(δ) versus log⁡δ using linear regression. To demonstrate that (22) correctly computes the fractal dimension, we introduce several additional definitions.

**Definition 1.** A function $f:[0,1]\to R^{d}$ is α-Hölder continuous if there exists C>0 such that $\|f(x)-f(y)\|\leq C|x-y{|}^{\alpha }$, for all x,y∈[0,1].

**Definition 2.** For a function $f:A\to R^{d}$, for A∈[0,∞), we define the graph to be ${\mathrm{Graph}}_{f}(A)=\{(t,f(t)):t\in A\}\subset R^{d+1}$.

**Proposition 1.** (*Proposition 4.14 in* [34]) $f:[0,1]\to R^{d}$ *is an* α-*Hölder continuous function. Then the Hausdorff dimension becomes*

(23) $$\dim\left({\mathrm{Graph}}_{f}([0,1])\right)\leq 1+(1-\alpha )\text{min}\left(d,\frac{1}{\alpha }\right).$$

Since f is α-Hölder continuous, there exists a constant C>0 such that, if s,t∈[0,1] with |s-t|≤ϵ, then

$$\left\|f(s)-f(t)\right\|\leq C\epsilon^{\alpha }.$$

For a given time series f(t), finding the dimension using (23) reduces to determining α. Assuming that $X_{t}(\epsilon )=\|f(t+\epsilon )-f(t)\|$ is independent of t, we further assume that $X_{t}(\epsilon )$ is ergodic in the mean. This implies that the sample time average $\overset{ˆ}{\mu }(\epsilon )=\left\langle X_{t}(\epsilon )\right\rangle$ converges to $\mu (\epsilon )=E\left(X_{t}(\epsilon )\right)$ in mean square, where ⟨⋅⟩ denotes a time average.

By the Chernoff bound, we have:

(24) $$\mathrm{Pr}\left(X_{t}(\epsilon )\leq (1+\eta )\mu (\epsilon )\right)\geq 1-e^{-\mu (\epsilon )\eta^{2}/\left(2+\eta \right)}.$$

For large η, with high probability,

‖f(t+ϵ)−f(t)‖≤(1+η)μ(ϵ).

If we can show that $\mu (\epsilon )\leq \beta \epsilon^{\alpha }$ for some constant β>0, it follows that:

(25) $$\left\|f(t+\epsilon )-f(t)\right\|\leq (1+\eta )\mu (\epsilon )\leq (1+\eta )\beta \epsilon^{\alpha },$$

which satisfies the α-Hölder continuity condition.

Since (1+η)β is a constant and we are only interested in α, we estimate α as the slope of the plot of $\log\overset{ˆ}{\mu }(\epsilon )=\log(\langle \|f(t+\epsilon )-f(t)\|\rangle )$ versus log⁡ϵ, determined through linear regression. Note that in (24), we assume $X_{t}(\epsilon )$ is independent of t, which may hold true when $X_{t}(\epsilon )$ values do not overlap for different t.

When applying the method to a dataset $\left\{x_{i}\right\},U(\delta )$ in (17) computes ⟨‖f(t+ϵ)-f(t)‖⟩, where ϵ=δΔt. The slope of log⁡U(δ) versus log⁡δ is equivalent to the slope of log⁡(⟨‖f(t+ϵ)-f(t)‖⟩) versus

logϵ=logδ+logΔt,

since log⁡Δt is a constant.

Finally, substituting α into (23) yields the FD as defined in (21). We have discussed various implementations of the temporal sampling method in Supplemental Information Section Appendix A.1 (Fig. S2). Additionally, we demonstrate that the temporal sampling algorithm accurately computes the Hausdorff dimension using the Koch curve and Brownian motion as examples (see Section Appendix A.2 in the Supplemental Information for further details).

The temporal sampling algorithm differs from the traditional divider method in two key ways. First, it utilizes the average distance, U(δ), rather than the total distance, L(δ), in the regression analysis. Second, the FD is calculated as the inverse of the slope in (22), rather than the slope itself. This algorithm offers significant advantages, particularly in terms of time and space efficiency. Computing U(δ) as defined in (17) does not require sorting or the use of additional hash tables, resulting in a time complexity of O(n/δ) and a space complexity of O(1). These features make the temporal sampling algorithm especially efficient in practical applications.

### 2.3. Drosophila melanogaster strains and husbandry

*Drosophila* stocks were maintained at room temperature on standard molasses-based food. For experiments, *Drosophila* alleles were reared at $25^{\circ }C$. The w1118 strain was used as the *wild-type* control. The *GluRIIA* mutant strain was obtained from the Bloomington *Drosophila* Stock Center (BL64202) [35]. The *Ada2b* mutant strain [29] was kindly provided by Dr. Jerry Workman from the Stowers Institute in Kansas City, Missouri. The *Dysbindin* mutant strain [20] was generously provided by Dr. Dion Dickman from the University of Southern California, Los Angeles, California. Equal numbers of male and female animals were used in the experiments.

### 2.4. Drosophila larval crawling data acquisition, pre-processing and statistics

*Drosophila* third instar larvae with the correct genotypes were rinsed with PBS to remove food and yeast residues, then allowed to habituate for 2 minutes at room temperature. Each larva was gently placed at the center of a 10 cm Petri dish (pre-warmed to room temperature) containing agar dyed with black food coloring. The free movement of the larva was recorded for 2 minutes. Larval movement was captured using OKIO S2 cameras (OKIO Labs) with OBS software (version 28.0.0, OBS Studio) at a resolution of 1920×1080 pixels and 30 frames per second (fps). A customized Python script was developed to automatically track the centroid of each larva and extract the movement path as a 2-dimensional time series, $\left(x_{i},y_{i}\right)$, where each point $\left(x_{i},y_{i}\right)$ represents the larva’s centroid location at time iΔt.

For data pre-processing, we normalize the trace by scaling. Let the larval trace be a 2-dimensional time series $\left(x_{i},y_{i}\right)$, where each point $\left(x_{i},y_{i}\right)$ represents the larva’s centroid location at time iΔt. Since the topology of a metric space remains unchanged under linear transformation, we set the starting point of the trace to (0,0) and scale the trace by $\eta =max\left(x_{max}-x_{min},y_{max}-y_{min}\right)$ so that it lies within [0,1]×[0,1]. The transformation is defined as:

(26) $$x^{'}=\frac{x-x_{\text{min}}}{\eta },\;y^{'}=\frac{y-y_{\text{min}}}{\eta }.$$

Scaling the trace does not affect the fractal dimension, as introducing a new scale variable $\epsilon^{'}=\epsilon /\eta$ results in $\log\epsilon^{'}=\log\epsilon -\log\eta$. Consequently, the slope of box-counting or temporal sampling remains unchanged. Alternatively, the average distance between consecutive video frames can be used for normalization, defined as $\eta =\frac{\sum_{i=1}^{n-1}\;\left\|x_{i+1}-x_{i}\right\|}{n-1}$.

Data were analyzed using the Python library developed for computing the fractal dimensions proposed in this study, available at https://github.com/wanglab-georgetown/fractal. An α-level of 0.05 was used to determine statistical significance. To validate assumptions, the normality of residuals from ANOVA tests across all datasets was assessed using the Shapiro-Wilk test. For datasets with normally distributed ANOVA residuals, Student’s t-test or ANOVA was applied. For datasets with nonnormal ANOVA residuals, the nonparametric Mann-Whitney U test was used.

## 3. Results

### 3.1. Compare the original box-counting and proposed algorithms

**Fig. 2.**
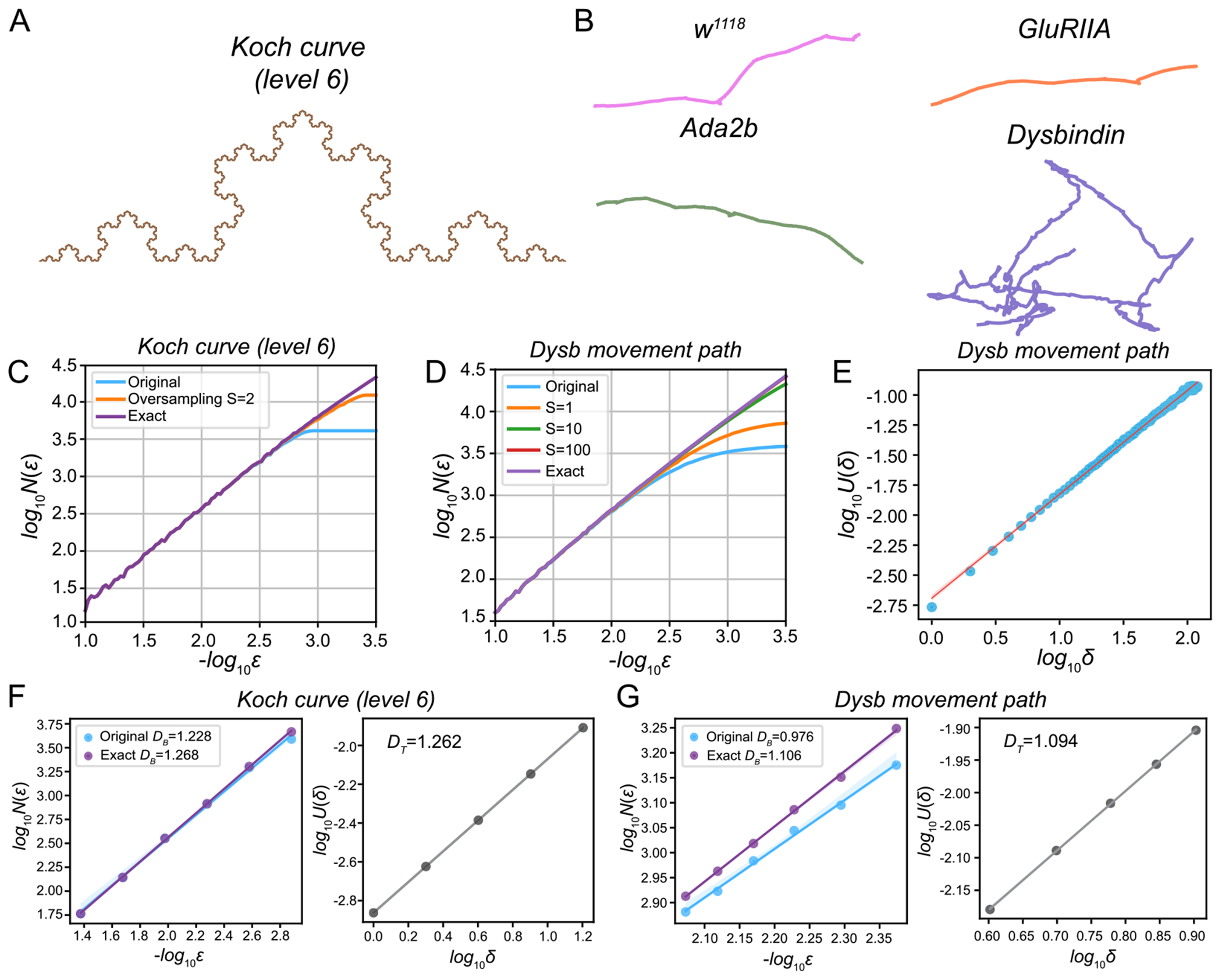
Comparisons of the original box-counting, oversampling, exact box-counting, and temporal sampling methods using the Koch curve and larval movement paths. **A**. The Koch curve (level 6). **B**. Representative *Drosophila* 3rd instar larval movement paths for the *wild-type* (*w1118*) and *GluRIIA, Ada2b*, and *Dysbindin* mutants. Normalized paths are shown. **C**. $\log_{10}N(\epsilon )$ vs $\log_{10}\epsilon$ plots for the Koch curve (level 6) are shown for the original box-counting (S=0), oversampling (S=2), and exact box-counting methods. **D**. $\log_{10}N(\epsilon )$ vs $\log_{10}\epsilon$ plots for the movement path of the *Dysbindin* mutant are shown for the original box-counting (S=0), oversampling (S=1,10,100), and exact box-counting methods. **E**. $\log_{10}U(\delta )$ vs $\log_{10}\delta$ plot (δ=1,…,100) for the movement path of the *Dysbindin* mutant is shown for the temporal sampling method. **F**. $\log_{10}N(\epsilon )$ (original and exact box-counting, left panel) and $\log_{10}U(\delta )$ (temporal sampling, right panel) are used for estimating FD of the level 6 Koch curve. **G**. $\log_{10}N(\epsilon )$ (original and exact box-counting, left panel) and $\log_{10}U(\delta )$ (temporal sampling, right panel) used for estimating FD for the movement path of the *Dysbindin* mutant.

We evaluate the performance of existing and proposed algorithms in calculating the fractal dimension using the Koch curve and real animal movement data (Fig. 2A and 2B). First, we compare the original box-counting algorithm (S=0), the oversampling algorithm (with =2), and the exact box-counting algorithm by calculating $\log_{10}N(\epsilon )$ versus $\log_{10}\epsilon$ for the Koch curve (level 6). We used 100 box sizes between $10^{-3.5}$ and $10^{-1}$, linearly spaced in $\log_{10}$ with an increment of 0.025, i.e., $\log_{10}\epsilon_{k}=-1-0.025k,k=0,\ldots ,99$. For large ϵ, all three algorithms yield the same N(ϵ) (Fig. 2C). However, as ϵ decreases, the original box-counting algorithm saturates at a higher ϵ compared to the oversampling algorithm. While the oversampling algorithm delays the saturation point, it eventually also saturates. In contrast, the exact box-counting algorithm achieves the highest N(ϵ) across all ϵ, attains the smallest saturation point, and converges to a line with a slope of −1, unlike the original box-counting algorithm, which converges to a slope of 0.

Next, we compare the original box-counting algorithm (S=0), the oversampling algorithm (with S=1,10,100), and the exact box-counting algorithm for calculating $\log_{10}N(\epsilon )$ versus $\log_{10}\epsilon$ for a representative movement path of the *Dysbindin* mutant in Fig. 2B. As S→+∞,N(ϵ) in the oversampling algorithm saturates more gradually as ϵ→0 (Fig. 2D). Moreover, the oversampling algorithm validates the exact box-counting algorithm by matching the boxes identified by both algorithms when S is large. To validate the temporal sampling algorithm using real data, we plot $\log_{10}U(\delta )$ versus $\log_{10}\delta$ for δ=1,…,100 using the movement path of the *Dysbindin* mutant (Fig. 2E). The results confirm that U(δ) follows the scaling law (20). When δ is small, the slope (α) is slightly higher, consistent with Proposition 2 in Supplemental Information Section Appendix A.2, which states that a small δ leads to an approximate $D_{T}=1/\alpha$ of 1.

Furthermore, we compare the original box-counting, exact box-counting, and temporal sampling algorithms in estimating the FD using the Koch curve (level 6, true FD = 1.262; Fig. 2F). All algorithms return similar FDs: original box-counting 1.228, exact box-counting 1.268, and temporal sampling 1.262. Due to saturation at approximately $\epsilon =10^{-2.8}$, the original box-counting algorithm underestimates the FD when using the selected set of ϵ. In contrast, the temporal sampling algorithm accurately determines the true FD. For the correlation sum method, the correlation integral C(ϵ) does not follow the scaling law $C(\epsilon )=\epsilon^{D}$ when ϵ is small. Consequently, the FD for the Koch curve calculated using Takens’ estimator [36] is slightly higher, at 1.292 (see Section Appendix A.3 in the Supplemental Information and Fig. S3 for details).

We also apply these algorithms to compute the FD for the movement path of the *Dysbindin* mutant (circles in Fig. 2G are used for the calculation). The FDs estimated are: original box-counting 0.976, exact box-counting 1.106, and temporal sampling 1.094. While the exact box-counting and temporal sampling algorithms produce similar FDs, the original box-counting algorithm underestimates the FD due to saturation in the selected range of ϵ. Additionally, the correlation sum method is highly sensitive to ϵ and produces highly variable FD estimates (Fig. S3). Therefore, we exclude the correlation sum method from further analyses in this study.

### 3.2. FD is anomalous when calculated using different scales

**Fig. 3.**
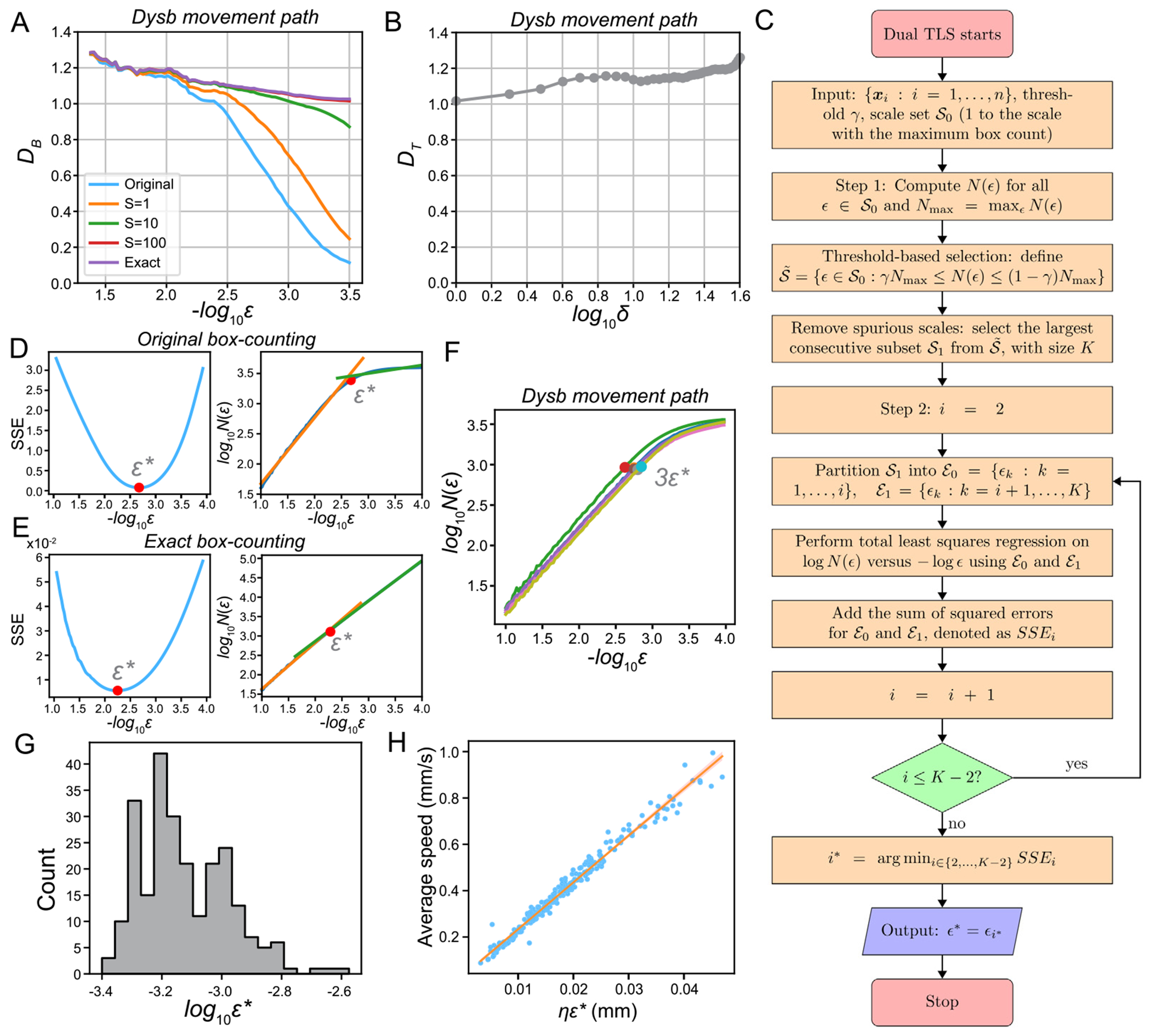
FD is anomalous across different measuring scales. **A**. The local FD D(ϵ) is estimated using a rolling window linear regression on the $\log_{10}N(\epsilon )$ vs log⁡ϵ plot with a window size of 15 for the *Dysbindin* mutant. The local FD D(ϵ) estimated through the original box-counting, oversampling (S=1, 10, 100), and exact box-counting methods are shown. **B**. The local FD estimated using a rolling window linear regression (=k,…,3k where k=1,…,40 and δ takes integers in 1, …, 120) for the *Dysbindin* mutant using the temporal sampling method. **C**. Flowchart showing the steps to identify the elbow point $\epsilon^{*}$ using the dual TLS method. **D**. SSE over log⁡ϵ plot (left panel) and TLS fitting (right panel) on the log⁡N(ϵ) plot. The elbow point $\epsilon^{*}$ identified through the dual TLS method with the original box-counting is shown in red. **E**. SSE over log⁡ϵ plot (left panel) and TLS fitting (right panel) on the log⁡N(ϵ) plot. The elbow point $\epsilon^{*}$ identified through the dual TLS method with the exact box-counting is shown in red. **F**. Elbow points $3\epsilon^{*}$ identified for movement paths of the *Dysbindin* mutant using the dual TLS method with the original box-counting. **G**. Histogram of $\epsilon^{*}$ identified for all the larval movement paths using the dual TLS method with the original box-counting. **H**. The average speed of larval movement shows a positive correlation with $\eta \epsilon^{*}$, which is the unnormalized box size in millimeter.

Next, we investigate in detail how the local FD D(ϵ) changes with scale ϵ in animal movement. We perform a rolling window linear regression on $\log_{10}N(\epsilon )$ versus $\log_{10}\epsilon$ with a window size of 15 to calculate the rolling FD for the *Dysbindin* mutant (Fig. 3A), where ϵ takes values $\log_{10}\epsilon_{k}=-1-0.025k,k=0,\ldots ,99$. The FD estimated by the original box-counting and oversampling algorithms converges to 0 at small scales, as 0 is the dimension of a finite set of points. While oversampling delays this convergence, it does not prevent it. In contrast, the exact box-counting algorithm, applied to a piecewise linear curve, converges to a dimension of 1 as ϵ→0. Interestingly, there are two scale ranges where FD remains almost constant across all three algorithms: one between $10^{-2.4}$ and $10^{-2.2}$, and another between $10^{-2.0}$ and $10^{-1.8}$. Each of these ranges may correspond to distinct regimes governed by different biological mechanisms underlying the crawling behavior.

Additionally, we analyze the local FD calculated from the relationship between $\log_{10}U(\delta )$ and $\log_{10}\delta$ using the temporal sampling algorithm (Fig. 3B). A rolling window linear regression is performed, where δ in each window takes values from k to 3k, with k ranging from 1 to 40. Each window maintains a uniform width in the logarithmic domain. At small values of δ, the dimension $D_{T}$ converges to 1, reflecting the linear movement of the animal over short time intervals. Similar to the results observed in the box-counting algorithms, there are two ranges of δ where $D_{T}$ remains nearly constant: between 5 and 9 (corresponding to δΔt≈0.2 seconds) and between 25 and 35 (corresponding to δΔt≈1 second). These ranges may suggest distinct neuronal mechanisms at the circuit level that regulate animal movement.

In summary, we observe that the dimension increases with larger spatial or temporal scales, indicating that the FD is anomalous and scale-dependent (Fig. 3A and 3B). Importantly, due to the anomalous nature of FD, both the local slope used for FD estimation and the overall shape of the curve provide valuable biological insights.

### 3.3. Determine the suitable scales for FD estimation

Given the scale dependency of FD (Fig. 3A and 3B), we explore methods to determine an appropriate set of scales for estimating FD, enabling fair comparisons across different genotypes. Because individual animals may exhibit varying movement speeds, applying identical scales across all animals can introduce biases when analyzing their diverse movement patterns. In mathematics and physics, a common practice is to manually select a linear segment of the log⁡N(ϵ) vs. log⁡ϵ plot for FD estimation [3]. However, this manual approach is not only subjective and prone to bias but also impractical for analyzing large-scale experimental datasets. To address these challenges, we propose a dual total least squares (TLS) method to systematically and automatically identify the inflection, or elbow point, in the log⁡N(ϵ) vs. log⁡ϵ plot. This method facilitates consistent alignment of movement paths across animals, ensuring scalability and reducing bias in FD estimation.

The algorithm consists of two main steps:

1. Select an initial set of scales.
2. Apply the dual TLS method to identify the elbow scale.

#### Step 1: Choosing an Initial Set of Scales

We first define a broad range of scales for the original box-counting algorithm, ensuring the number of boxes N(ϵ) spans from 1 to its saturation value. Let this initial scale set be denoted as $S_{0}$. For instance, we can set $S_{0}=\left\{10^{-0.025k}:k=0,\ldots ,400\right\}$. Using the original box-counting algorithm, we calculate N(ϵ) for each ϵ in $S_{0}$. Since N(ϵ) is often noisy and very small when ϵ is small, and remains constant at large ϵ, we aim to identify a subset of scales $S_{1}$ where N(ϵ) increases approximately monotonically. We propose two methods to refine $S_{1}$:

- Method 1, Threshold-Based Selection: Let $N_{max}={max}_{\epsilon }\;N(\epsilon )$ and γ be a percentage threshold. We define $S_{1}$ as:
$$\tilde{S}=\left\{\epsilon \in S_{0}:\gamma N_{\text{max}}\leq N(\epsilon )\leq (1-\gamma )N_{\text{max}}\right\}.$$
- Method 2, Regression-Based Selection: We perform linear regression on $\log_{10}N(\epsilon )$ versus $-\log_{10}\epsilon$ for $\epsilon \in S_{0}$ between 1 and the first ϵ that achieves $N_{\text{max}}$. Let the regression line be $-a\log_{10}\epsilon +b$. We then define $S_{1}$ as the set of scales above the regression line, i.e.,
$$\tilde{S}=\left\{\epsilon \in S_{0}:N(\epsilon )>-a{\text{log}}_{10}\epsilon +b\right\}.$$

In both methods, we retain the largest consecutive subset of $\tilde{𝒮}$, eliminating spurious scales that fluctuate above and below the threshold line. The resulting scale set is denoted as $S_{1}=\left\{\epsilon_{k}:k=1,\ldots ,K\right\}$, where K represents the number of scales in $S_{1}$.

#### Step 2: Identifying the Elbow Point

Using the scale set $S_{1}$, we partition it into two subsets for each i∈{2,…,K-2}:

$${ℰ}_{0}=\left\{\epsilon_{k}:k=1,\ldots ,i\right\},\;{ℰ}_{1}=\left\{\epsilon_{k}:k=i+1,\ldots ,K\right\}.$$

We apply TLS [37] to log⁡N(ϵ) versus -log⁡ϵ on both ${ℰ}_{0}$ and ${ℰ}_{1}$, adding the sum of squared errors (SSE) for each partition, denoted as $SSE_{i}$.

The elbow point corresponds to the partition that minimizes ${\mathrm{SSE}}_{i}$:

$$i^{\text{*}}=\text{arg}\underset{i\in \{2,\ldots ,K-2\}}{\text{min}}SSE_{i},\;\epsilon^{\text{*}}=\epsilon_{i^{\text{*}}},$$

where $\epsilon^{*}$ represents the scale at the elbow point. A flowchart illustrating the dual TLS algorithm is provided in Fig. 3C.

Fig. S4A illustrates an example of applying Method 1 to the *Dysbindin* movement path shown in Fig. 2B, using original box-counting method with a threshold of γ=0.01. The y-axis represents $\log_{10}N(\epsilon )$. In contrast, Fig. S4B displays the corresponding N(ϵ) values. Fig. S4C demonstrates the application of Method 2 to the same *Dysbindin* movement path using original box-counting. Additionally, we computed FD using Method 1 with thresholds γ=0.01,0.02,0.03,0.05, as well as Method 2 (Regression), across all 230 animal movement paths (Fig. S4D). The results reveal:

- Both the exact box-counting and temporal sampling algorithms yield statistically similar FD estimates across all values of γ in Method 1 and are consistent with estimates obtained using Method 2. For Method 1(γ=0.01) compared to Method 1 (γ=0.02,0.03,0.05) and Method 2, the p-values from the Mann-Whitney U test are 0.916, 0.806, 0.789, and 0.732 for exact box-counting, and 0.705, 0.601, 0.453, and 0.314 for temporal sampling (Fig. S4D).
- For the original box-counting algorithm, FD estimates using Method 1 are statistically similar across different γ values but show significant differences when compared to estimates obtained using Method 2. For Method 1(γ=0.01) compared to Method 1 (γ=0.02,0.03,0.05) and Method 2, the p-values from the Mann-Whitney U test are 0.451, 0.599, 0.331, and $5.873\times 10^{-4}$ (Fig. S4D).

These findings indicate that the original box-counting algorithm is sensitive to the choice of scale range, whereas the proposed exact box-counting and temporal sampling algorithms demonstrate robustness to this selection. Based on the consistency of FD estimates across different values of γ, we adopt Method 1 with γ=0.01 for all subsequent analyses.

Fig. 3D and 3E illustrate that the TLS fits on ${ℰ}_{0}$ and ${ℰ}_{1}$ effectively identify the elbow point $\epsilon^{*}$ for the movement path of the *Dysbindin* mutant using both the original and exact box-counting algorithms. Notably, the SSE obtained from the original box-counting algorithm is significantly higher than that from the exact box-counting method. Additionally, the slope calculated using the exact box-counting method remains relatively stable across different scales, in contrast to the variability observed with the original box-counting algorithm. However, the exact box-counting method’s sensitivity to fluctuations in N(ϵ) can allow noise to dominate the SSE, potentially leading to inaccurate estimates of $\epsilon^{*}$. To address this issue, we leverage the $\epsilon^{*}$ derived from the original box-counting algorithm to determine a scaled value, $\xi \epsilon^{*}$, for the exact box-counting method by multiplying $\epsilon^{*}$ by a constant ξ. Analysis of real larva movement data reveals that the $\epsilon^{*}$ for the exact box-counting algorithm is approximately 2.66 times that of the original box-counting method. Based on this observation, we adopt ξ=3 to fine-tune the selection of an optimal $\epsilon^{*}$ for estimating FD.

For the temporal sampling method, U(δ) can be interpreted as the box size (see Supplemental Information Section Appendix A.4). For a given $\epsilon^{*}$, the corresponding $\delta^{*}$ is determined as the minimum δ such that U(δ) satisfies $U(\delta )\geq \xi \epsilon^{*}$, i.e.,

$$\delta^{\text{*}}=\text{inf}\left\{\delta :U(\delta )\geq \xi \epsilon^{\text{*}}\right\}.$$

To further evaluate the reliability of identifying elbow points, we applied the dual TLS method to multiple movement paths of the *Dysbindin* mutant. The results confirm that $\epsilon^{*}$ can be effectively identified using the dual TLS method with the original box-counting (circles in Fig. 3F represent $3\epsilon^{*}$). Furthermore, we analyzed the distribution of $\epsilon^{*}$ values obtained through the dual TLS method with original box-counting across all larval movement paths. This analysis revealed a broad distribution (Fig. 3G), with $\epsilon^{*}$ values exhibiting approximately a fivefold variation. To investigate the biological significance of the optimized $\epsilon^{*}$ used for aligning movement paths across different animals, we examine its relationship with average movement speed. Interestingly, we find a strong positive linear correlation between the unnormalized $\eta \epsilon^{*}$ and the average speed of larval movement (Pearson correlation coefficient: 0.989, p-value: $7.51\times 10^{-204}$; Fig. 3H). This finding highlights the necessity of accounting for movement speed when aligning movement paths for FD comparisons across genotypes. When animals move at different speeds, using the same set of scales for FD estimation and comparison can introduce biases. Instead, either the path or the box size should be normalized by the average speed to ensure fair comparisons across animals. Moreover, average speed is proportional to the average distance between consecutive video frames $\left(\frac{\sum_{i=1}^{n-1}\left\|x_{i+1}-x_{i}\right\|}{n-1}\right)$. On average, we find that $\eta \epsilon^{*}$ is approximately 1.34 times the average distance. Selecting $3\eta \epsilon^{*}$ as the optimal scale corresponds to roughly four times the average distance. This observation also indicates that the average distance can be used as a normalization factor for each animal’s movement path.

Given $\epsilon^{*}$, it is important to appropriately select a subset of scales $S_{2}$ around $\epsilon^{*}\left(\epsilon^{*}\in S_{2}\right)$ for FD estimation, ensuring that N(ϵ) monotonically decreases within $S_{2}$. For example, Fig. 2C and 2D demonstrate cases where N(ϵ) does not exhibit monotonic behavior with respect to ϵ. To achieve monotonicity, we define $\epsilon_{k+1}=\lambda_{k}\epsilon_{k}$, where $\lambda_{k}\in N$ is a positive integer. Since $\lambda_{k}^{d}$ boxes of size $\epsilon_{k}$ correspond to one box of size $\epsilon_{k+1}$, this guarantees that $N\left(\epsilon_{k}\right)\geq N\left(\epsilon_{k+1}\right)$. The smallest allowable value for $\lambda_{k}$ is 2. One potential set of scales is:

(27) $$\left\{\epsilon^{\text{*}}2^{k}:k=0,\ldots ,K-1\right\}.$$

Similarly, for temporal sampling, we define $\delta_{k+1}=\lambda_{k}\delta_{k}$, where $\lambda_{k}\in$ N is a positive integer. If $\lambda_{k}=2$, one possible set of scales is:

(28) $$\left\{\delta^{\text{*}}2^{k}:k=0,\ldots ,K-1\right\}.$$

As a specific case where K=2, the local D(ϵ) at any scale ϵ can be calculated as:

(29) $$D(\epsilon )=-\frac{\text{log}N(2\epsilon )-\text{log}N(\epsilon )}{\text{log}2\epsilon -\text{log}\epsilon }.$$

### 3.4. The effect of box placement on FD

**Fig. 4.**
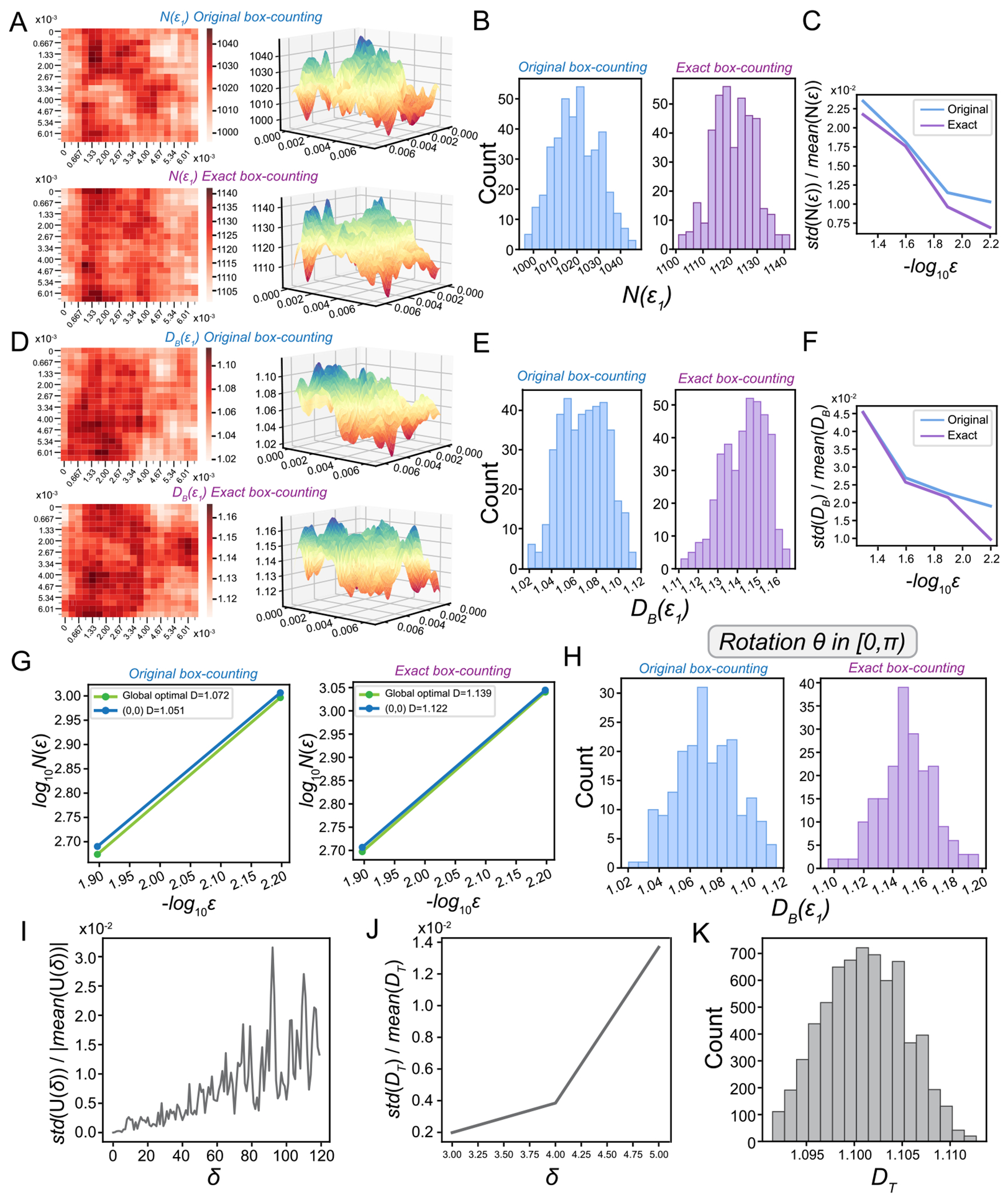
The impact of box placement on FD estimation. **A**. $N\left(\epsilon_{1}\right)$ calculated using the original box-counting (upper panels) and exact box-counting (lower panels) for the *Dysbindin* mutant by shifting the origin of box-counting grid ($o_{x},o_{y}$) to each of the 400 grid point in a $\left[0,\epsilon_{1}\right)\times \left[0,\epsilon_{1}\right)$ box $\left(\epsilon_{1}=3\epsilon^{*}\right)$. *N*($\epsilon_{1}$) is shown in 2D (left panels) and 3D (right panels) heat maps. **B**. The histogram of $N\left(\epsilon_{1}\right)$ calculated using the original box-counting (left panel) and exact box-counting (right panel) for the *Dysbindin* mutant by shifting the origin of box-counting grid $\left(o_{x},o_{y}\right)$ to each of the 400 grid point in a $\left[0,\epsilon_{1}\right)\times \left[0,\epsilon_{1}\right)$ box. **C**. The variations (std(N(ϵ))/mean(N(ϵ)) of N(ϵ) across different ϵ are shown for the original and exact box-counting methods. **D**. $D_{B}\left(\epsilon_{1}\right)$ calculated by the original box-counting (upper panels) and exact box-counting (lower panels) for the *Dysbindin* mutant by shifting the origin of box-counting grid ($o_{x},o_{y}$) to each of the 400 grid point in a $[0,\epsilon_{1})\times \left[0,\epsilon_{1}\right.$) box $\left(\epsilon_{1}=3\epsilon^{*}\right).D_{B}\left(\epsilon_{1}\right)$ is shown in 2D (left panels) and 3D (right panels) heat maps. **E**. The histogram of $D_{B}\left(\epsilon_{1}\right)$ calculated using the original box-counting (left panel) and exact box-counting (right panel) for the *Dysbindin* mutant by shifting the origin of box-counting grid ($o_{x},o_{y}$) to each of the 400 grid point in a $[0,\epsilon_{1})\times \left[0,\epsilon_{1}\right.$ box. **F**. The variations $\left(stdD_{B}(\epsilon )/mean\left(D_{B}(\epsilon )\right)\right.$ of $D_{B}(\epsilon )$ across different ϵ are shown for the original and exact box-counting methods. **G**. The global optimal $N^{*}(\epsilon )$ and N(ϵ) calculated from a fixed grid placed at (0, 0) through the original (left panel) and exact (right panel) box-counting methods. **H**. The histogram of $D_{B}\left(\epsilon_{1}\right)$ across different rotations (angle θ,200 uniformly spaced values between 0 and *π*) of a grid positioned at the origin (0, 0) calculated by the original (left panel) and the exact (right panel) box-counting methods. **I**. The variation of U(δ)(std(U(δ))/mean(U(δ))) across different start point o in the time series using the temporal sampling method. **J**. The variation of $D_{T}(\delta )\left(std\left(D_{T}(\delta )\right)/mean\left(D_{T}(\delta )\right)\right)$ across different start points o at δ=3,4,5 calculated by the temporal sampling method. **K**. The histogram of $D_{T}(4)(\delta =4)$ over 6720 starting points in the time series is shown.

While using a fixed grid of cubes rather than optimized cubes does not change $D_{B}$ in (3) if ϵ→0 [5], $D_{B}(\epsilon )$ may depend on the placement of the grid when a finite ϵ is applied to biological questions. We first investigate the impact of the origin of the grid $\left(o_{x},o_{y}\right)$ on N(ϵ) and $D_{B}(\epsilon )$ using the movement path of the *Dysbindin* mutant. We restrict $\left(o_{x},o_{y}\right)$ to be in box [0,ϵ)×[0,ϵ), as grid is a periodical structure. We choose $\epsilon_{1}=3\epsilon^{*}$, where $\epsilon^{*}$ is the elbow point from Section 3.2, and test the variation in N(ϵ) over $\epsilon =2^{k}\epsilon_{1},k=0,1,2,3$. For each ϵ, we uniformly sample [0,ϵ)×[0,ϵ) as a 20×20 grid and compute N(ϵ) by shifting the origin of box-counting grid to each of the 400 grid point (Fig. 4A–4C). We find that N(ϵ) has many local minima, posing challenges for optimization algorithms to identify the global minimum (Fig. 4A). Moreover, N(ϵ) achieves its minimum at different origins for different ϵ. We find the original box-counting has a greater variation in box counts across 400 placements at $\epsilon_{1}\left(\mathrm{std}\left(N\left(\epsilon_{1}\right)\right)/\mathrm{mean}\right.\left.\left(N\left(\epsilon_{1}\right)\right),1.03\%\right)$ than the exact box-counting method (0.69%, Fig. 4B). Additionally, the variation of N(ϵ) increases with ϵ, indicating a greater ϵ leads to a higher uncertainty in box counts (Fig. 4C).

Next, we directly investigate the variation of $D_{B}$ over ϵ with different placement of the grid (Fig. 4D–4F). We use the same $\epsilon =2^{k}\epsilon_{1},k=0,1,2,3$ and 400 shifts to calculate N(ϵ). For each ϵ, we use N(ϵ) at ϵ,2ϵ to estimate $D_{B}(\epsilon )$ as in (29). The variation of $D_{B}(\epsilon )$ increases with ϵ, suggesting that a greater ϵ leads to higher uncertainty in $D_{B}$, and thus, small scales ϵ should be used for FD estimation (Fig. 4F). However, the selected set of scales should not be smaller than $\epsilon^{*}$ as described in Section 3.2. We find that the original box-counting not only underestimates $D_{B}$, but also has higher uncertainty compared to the exact box-counting (Fig. 4E and 4F).

Additionally, we perform a global optimization to find the global minimum of N(ϵ) by initiating from each of the 400 grid placements and selecting the minimum value among the 400 runs. We compare the $D_{B}$ estimated using global optimized $N^{*}(\epsilon )$ with the N(ϵ) calculated using a fixed grid at origin (0,0). For both the original and exact box-counting algorithms, the global optimal $N^{*}(\epsilon )$ is almost parallel to N(ϵ) calculated from a fixed grid placed at (0,0), resulting in very close $D_{B}$ estimates (Fig. 4G). $D_{B}$ values obtained from various grid origins (Fig. 4E) may randomly fluctuate around the $D_{B}$ value calculated through the globally optimized $N^{*}(\epsilon )$.

Furthermore, we investigate how the rotation of the grid affect FD estimation. We rotate the grid at the origin (0,0), by angle θ, where θ takes one of the 200 uniformly spaced values between 0 and π. We find the original box-counting has a slightly higher variation in $D_{B}\left(\epsilon_{1}\right)$ than the exact box-counting method, with $std\left(D_{B}\left(\epsilon_{1}\right)\right)/mean\left(D_{B}\left(\epsilon_{1}\right)\right)1.82\%$ vs 1.53% (Fig. 4H). It is worth noting that in real experimental data, animal movement paths typically start with arbitrary orientations, and the box grid is randomly positioned over these paths. From a practical standpoint, when assessing the FD across different genotypes, this inherent randomness in grid placement is factored into the statistical analysis framework. With our experimental dataset comprising a substantial number of movement paths for each genotype, it allows for direct statistical analysis of the estimated $D_{B}$ using a fixed grid. Therefore, choosing a less optimal N(ϵ) at a fixed origin reduces computational demands while still yielding similar results in FD comparison.

An advantage of the temporal sampling algorithm over the box-counting algorithm is that U(δ) and hence $D_{T}$ are invariant to curve rotation, eliminating one source of uncertainty. However, in (17), U(δ) is computed starting from the first point. We investigate how the starting point affect FD estimation. If we start from the o-th point instead o∈{1,…,δ-1}, i.e.,

(30) $$U(\delta ,o)=\frac{1}{m(\delta ,o)}\overset{m(\delta ,o)}{\underset{i=1}{\sum }}\left\|x_{i\delta +o}-x_{(i-1)\delta +o}\right\|,$$

where

(31) $$m(\delta ,o)=\left\lfloor \frac{n-o}{\delta }\right\rfloor .$$

For each δ∈{1,…,120}, we compute U(δ,o) using (30) for each o and compute std(U(δ))/mean(U(δ)). Similar to the box-counting algorithm, the normalized variation in U(δ) has a trend to increase with δ (Fig. 4I). For a given δ, we compute $D_{T}$ using 𝒟(δ)={δ,…,2δ} and for each $\delta_{k}\in 𝒟(\delta )$ we calculate U(δ) for each $\delta_{k}$ offset, which results in (2δ)!/(δ-1)! total 𝒟(δ) for each δ. We find the variation in $D_{T}$ increases with δ (δ=3,4,5), which suggests that small δ should be used to estimate U(δ) to reduce variation in $D_{T}$ (Fig. 4J). The histogram of $D_{T}(4)(\delta =4)$ over 6720 initial offsets is shown (Fig. 4K). For the temporal sampling algorithm, we find the normalized standard deviation of $D_{T}$ is around 0.4% when $\delta^{*}$ (see Section 3.3) is used in estimating $D_{T}$.

### 3.5. FD estimated for movement paths in different genotypes

**Fig. 5.**
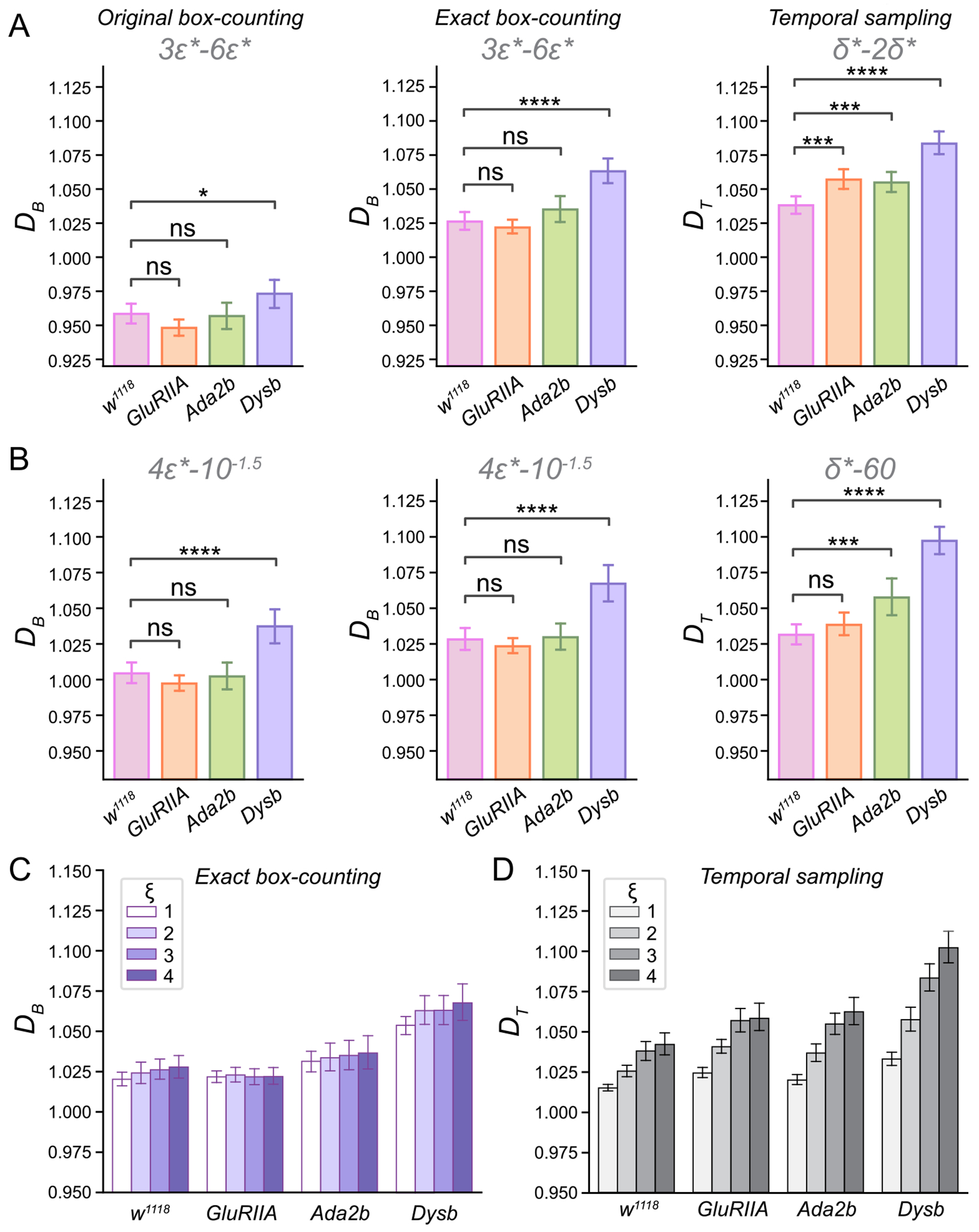
Comparisons of FD across different genotypes. **A**. FD calculated through different algorithms using scales $\left\{3\epsilon^{*},4.5\epsilon^{*},6\epsilon^{*}\right\}$ (box-counting) and $\left\{\delta^{*},\ldots ,2\delta^{*}\right\}$ (temporal sampling) for the *wild-type* (n=54) and mutant strains of *GluRIIA* (n=76), *Ada2b* (n=50), and *Dysbindin* (*Dysb*, =50). *Mean* ±95% confidence interval, ns not significant, *p < 0.05, **p < 0.01, ***p < 0.001, ****p < 0.0001, Mann-Whitney U test. **B**. FD calculated through different algorithms using scales between $4\epsilon^{*}$ and $10^{-1.5}$ with stepsize 0.025 in $\log_{10}\epsilon$ (box-counting) and $\delta \in \left\{\delta^{*},\ldots ,60\right\}$ (temporal sampling) for the *wild-type* (n=54) and mutant strains of *GluRIIA* (n=76), *Ada2b* (n=50), and *Dysbindin* (*Dysb*, =50). Mean±95% confidence interval, ns not significant, *p < 0.05, **p < 0.01, ***p < 0.001, ****p < 0.0001, Mann-Whitney U test. **C**. FD calculated using different scale $\xi \epsilon^{*}$ through the exact box-counting method for different genotypes. **D**. FD calculated using different scale $\xi \epsilon^{*}$ through temporal sampling method for different genotypes.

We compare the original box-counting, exact box-counting, and temporal sampling algorithms for estimating the FD of movement paths in *wild-type* and mutant strains of *GluRIIA, Ada2b*, and *Dysbindin*. For the box-counting methods, we estimate $D_{B}$ using the scales $\left\{3\epsilon^{*},4.5\epsilon^{*},6\epsilon^{*}\right\}$, where $\epsilon^{*}$ is identified at the elbow point using the dual TLS method. For the temporal sampling algorithm, we determine $\delta^{*}=inf\left\{\delta :U(\delta )\geq 3\epsilon^{*}\right\}$ and use the range $\left\{\delta^{*},\ldots ,2\delta^{*}\right\}$ to estimate $D_{T}$. The nonparametric Mann-Whitney U test was used to calculate p-values. Using the chosen scales, we computed the local FD with the three algorithms (Fig. 5A). FD estimates from the original box-counting method were consistently smaller than those obtained from the other two algorithms. The temporal sampling algorithm provided similar but slightly higher FD estimates compared to the exact box-counting method. Across all FD algorithms, *Dysbindin* mutants consistently exhibited higher FDs than the *wild-type* (original box-counting: $p=1.803\times 10^{-2}$; exact box-counting: $p=1.801\times 10^{-9}$; temporal sampling: $p=1.235\times 10^{-11}$). Similarly, *GluRIIA* ($p=6.528\times 10^{-4}$) and *Ada2b* ($p=5.313\times 10^{-4}$) mutants showed higher FDs estimated by the temporal sampling algorithm compared to the *wild-type*. However, FDs calculated by the box-counting methods did not show significant differences from the *wild-type* (*GluRIIA*: p=0.304; *Ada2b*: p=0.251 using exact box-counting). These findings suggest that different algorithms may vary in sensitivity when detecting differences in the tortuosity of animal movement paths.

We also computed the FD using larger scales (*ϵ* between $4\epsilon^{*}$ and $10^{-1.5}$ with a stepsize of 0.025 in $\log_{10}\epsilon$) for the box-counting algorithms and $\delta \in \left\{\delta^{*},\ldots ,60\right\}$ for the temporal sampling algorithm (Fig. 5B). Consistent with the observations at smaller scales ($3\epsilon^{*}$), *Dysbindin* mutants exhibited higher FDs than the *wild-type* across all algorithms (original box-counting: $p=2.678\times 10^{-6}$; exact box-counting: $p=5.869\times 10^{-8}$; temporal sampling: $p=5.576\times 10^{-14}$).

*DTNBP1*, the human homolog of the *Drosophila Dysbindin* gene, is known to affect behaviors in a sex-dependent manner [38]. Interestingly, the FD calculated using the exact box-counting method was slightly higher in male than in female *Dysbindin* mutants (p=0.0908, Fig. S5), consistent with observations of *DTNBP1* showing sex-dependent effects on cognitive functions in mice and humans [38].

To explore variations in local FD as a function of the starting scale $\xi \epsilon^{*}$, we varied ξ from 1 to 4 (Fig. 5C). Using the exact box-counting algorithm, we observed that the FD increased with ξ in the *wild-type*, while remaining relatively stable for the *GluRIIA* and *Ada2b* mutants. This suggests that the *GluRIIA* and *Ada2b* mutants may exhibit distinct local dimensional structures compared to the *wild-type*. For *Dysbindin* mutants, the FD initially increased with ξ before stabilizing, consistently remaining higher than the *wild-type* across all ξ values.

Using the temporal sampling algorithm, the FD increased with ξ for all genotypes. However, the FDs for the *wild-type* and *GluRIIA* mutants stabilized when ξ=4. This indicates that the temporal sampling algorithm may capture different FD structures than the box-counting algorithm, as both $D_{B}$ and $D_{T}$ serve as upper bounds for $D_{H}$.

Given the anomalous nature of FD, careful selection of scales and algorithms is crucial when comparing conditions. Overall, FD estimates across various scales may reveal motor movement patterns at different spatial and temporal scales, providing critical insights into the circuit mechanisms underlying diverse animal behaviors.

## 4. Discussion

Fractal dimension is an effective quantitative tool for analyzing the complexity of animal movement paths, particularly for large-scale data analysis. We propose using oversampling, exact box-counting, and temporal sampling algorithms for robust and reliable FD estimation (Fig. 1 and Fig. 2). To address the heterogeneity of FD across different scales, we introduce a dual total least squares method for identifying elbow points. This approach enables effective alignment of movement paths from various genotypes, ensuring fair comparisons. By leveraging these elbow points, we select appropriate scales for FD computation across animals and genotypes (Fig. 3). We systematically examined the impact of grid placement in box-counting and the choice of starting time in temporal sampling on FD estimation (Fig. 4). Additionally, we explored generalized dimensions and found that box-counting methods are well-suited for capturing the complexity of animal movement paths (see Supplemental Information Section Appendix A.4 and Fig. S6). These new algorithms and insights enhance the utility of FD for quantitative comparisons of motor phenotypes in animals. They provide valuable frameworks for investigating genotypic-phenotypic connections, offering profound insights into the genetic regulation of motor functions (Fig. 5).

It is important to note that the oversampling and exact box-counting algorithms utilize the piecewise linear curve, potentially extending the range of ϵ suitable for estimating the FD by lowering the threshold at which N(ϵ) stabilizes. This approach reduces the variability of N(ϵ) and the estimated box-counting dimension $D_{B}$, which can be influenced by the grid’s origin placement and rotations. However, selecting an arbitrarily small ϵ is not feasible. As ϵ approaches zero, the estimated FD on the piecewise linear curve converges to 1. The minimum allowable ϵ is constrained by the average spacing between the original points $x_{i}$, defined as $\sum_{i=1}^{n-1}\;\left\|x_{i+1}-x_{i}\right\|/(n-1)$. Consequently, ϵ should not be smaller than $\epsilon^{*}$, which is identified using the dual TLS method.

The temporal resolution of the animal path directly influences the resolution of both box-counting and temporal sampling algorithms. A lower frame rate increases the distance between adjacent video frames, leading to earlier saturation of the original box-counting method at larger scales. For the temporal sampling algorithm, a reduced frame rate removes data points for small δ from the log⁡U(δ) versus log⁡δ plot, reducing the algorithm’s ability to accurately estimate the FD at smaller time intervals. As shown in Fig. 3A, two distinct scale ranges emerge where the FD remains relatively constant across all three algorithms: one corresponding to a time interval of approximately 0.2 seconds and another to 1 second. Capturing these regimes requires a sufficiently high video frame rate to resolve time intervals within these scales. An insufficient frame rate may obscure these FD patterns, limiting the ability to analyze movement complexity at finer temporal resolutions.

Numerous challenges limit the application of FD in addressing biological questions. To overcome the constraints imposed by finite box sizes for quantifying FD in biological systems, we explore the use of oversampling and exact box-counting in the spatial domain, along with temporal sampling in the temporal domain. Much of the prior skepticism regarding FD arises from studies relying on simulation data rather than real experimental datasets. In contrast, we leverage high-resolution larval crawling data to investigate how genetic mutations influence animal behavior through FD. Unlike previous studies that typically calculate FD as an absolute value for specific conditions, our study compares FD across genotypes, treating it as a quantitative measure. This necessitates a consistent definition of FD across various traces, emphasizing the importance of robust FD estimation.

FD reflects the degrees of freedom of a dynamic system, with higher FD values potentially indicating decoupling of previously coupled components, resulting in increased complexity. This rationale suggests that larvae with impairments at the NMJ or circuit level may exhibit abnormal movement behaviors, leading to higher FD values. Analysis of larval movement data reveals that movement appears linear at the smallest scales but exhibits increasing FD at larger scales. This variation in FD across scales provides insights into animal behavior and brain circuitry at different temporal resolutions. Interestingly, the relationship between the Hurst exponent H (a measure of long-term memory in time series) and FD, defined by D+H=d+1 [39], highlights FD as a potential measure of the long-term memory of a time series. This positions FD as a valuable tool for understanding the temporal dynamics of animal behavior. While mathematical memory may not directly equate to biological memory, FD could be mathematically linked to neuronal memory mechanisms. Beyond a single FD value, the entire curve of N(ϵ) versus ϵ could reveal valuable information about underlying circuit functions.

Our findings indicate that FD for *Dysbindin* mutants is consistently higher than for *wild-type* control animals across all estimation methods, suggesting impaired movement behaviors due to peripheral or central synaptic and circuit deficits (Fig. 5). Conversely, *GluRIIA* mutants show an increase in FD compared to controls when temporal sampling with small scales is used but exhibit no FD change with the original or exact box-counting algorithms (Fig. 5A and Fig. 5B). This implies that homeostatic plasticity mechanisms, such as PHP, may compensate for motor function deficits in *GluRIIA* mutants, preserving relatively normal motor function. Interestingly, Ada2b mutants exhibit higher FD at both small and large scales when estimated using the temporal sampling algorithm, suggesting moderate motor deficits likely caused by impaired homeostatic plasticity due to mutations in the *Ada2b* gene (Fig. 5A and Fig. 5B). Consistent with prior findings of sex differences in *Dysbindin* mutants, males show higher FD than females, indicating that motor function may be regulated in a sex-specific manner by the *Dysbindin* gene (Fig. S5).

Overall, our study demonstrates that FD is an effective metric for comparing movement paths across genotypes, providing valuable insights into the genetic and neuronal regulation of motor behaviors. Using *Drosophila* larval movement data, we establish FD as a robust tool for analyzing the complexity of animal movement and its underlying mechanisms. However, it is important to emphasize that an increase in FD does not necessarily reveal the cellular or molecular mechanisms driving changes in behavioral complexity, highlighting the need for further investigation using complementary biological approaches. Despite this limitation, the FD estimation methods developed in this study can be broadly applied to investigate brain functions in other species, as well as to explore complexity, scaling properties, and self-similarity in theoretical and engineering domains. Beyond basic and theoretical research, the proposed FD analysis tools hold promise for practical applications, such as identifying genetic markers for motor impairments in large-scale human patient datasets and developing diagnostic tools for neurological disorders associated with motor function deficits.

## 5. Conclusion

In this study, we developed and evaluated advanced algorithms for estimating the fractal dimension of animal movement, including the exact box-counting and temporal sampling methods. These methods demonstrated robust performance in capturing complex movement patterns across varying spatial and temporal scales. Our findings, particularly the observed differences in FD among *Drosophila* genotypes, highlight the utility of FD as a quantitative metric for analyzing movement behaviors and understanding the underlying neuronal mechanisms. The ability to resolve scale-dependent dynamics underscores the critical importance of selecting appropriate scales and algorithms tailored to specific biological or physical contexts. The methodologies and insights presented in this work extend beyond biological applications, offering significant potential for engineering fields that analyze complex, dynamic systems. By illustrating how FD estimation can reveal meaningful patterns in biological systems, this work provides a foundation for applying these methods to diverse challenges in engineering and data science. As FD continues to serve as a powerful tool for quantifying complexity, further interdisciplinary research can harness its potential to address critical problems in both natural and engineered systems.

## Supplementary Material

1

## Data Availability

The Materials and Methods section provides detailed descriptions of all mathematical models and algorithms. A Python library for computing fractal dimensions is available at https://github.com/wanglab-georgetown/fractal.

## Glossary

FD: Fractal Dimension
NMJ: Neuromuscular Junction
PHP: Presynaptic Homeostatic Potentiation
Dysbindin: Dystrobrevin-binding Protein
DPC: Dystrophin-associated Protein Complex
BLOC: Biogenesis of Lysosome-related Organelles Complex
D_B_: Fractal dimension estimated using the box-counting method
D_T_: Fractal dimension estimated using the temporal sampling method
