# Supplementary material for: Exact box-counting and temporal sampling algorithms for fractal dimension estimation with applications to animal behavior analysis
Source: Results Eng. Author manuscript; Available in PMC 2025 Apr 14. (PMC11996205; doi:10.1016/j.rineng.2024.103755)
Supplement: 1 [file NIHMS2069134-supplement-1.pdf]

Exact Box-Counting and Temporal Sampling Algorithms for Fractal Dimension Estimation with Applications to Animal Behavior Analysis

Tao Cui[1,], Tingting Wang[1,]

[a]*Department of Pharmacology and Physiology , Georgetown University Medical Center, Washington , 20007, D.C., USA*

Email addresses: `tc936@georgetown.edu` (Tao Cui), `tw652@georgetown.edu` (Tingting Wang)

URL: `https://orcid.org/0000-0002-1004-4491` (Tao Cui), `https://orcid.org/0000-0001-5114-6737` (Tingting Wang)

[1]To whom correspondence should be addressed

Appendix A. Supplemental Materials

Appendix A.1. Variants of the temporal sampling algorithm

For a one-dimensional Brownian motion, $f(t+\epsilon) - f(t)$ is normally distributed with a mean of 0 and a standard deviation of $\sigma(\epsilon) = \sqrt{\epsilon}$. Instead of using the first-order moment to estimate $\sigma(\epsilon)$, we can estimate the standard deviation using any absolute moment of order p as

$$\sigma(\epsilon) = \gamma \sqrt[p]{\langle \|f(t+\epsilon) - f(t)\|^p \rangle}, \tag{A.1}$$

where γ is a constant. Using this approach and applying Hölder's Inequality, we get

$$\langle \|f(t+\epsilon) - f(t)\| \rangle \leq \sqrt[p]{\langle \|f(t+\epsilon) - f(t)\|^p \rangle}, \tag{A.2}$$

for $p \geq 1$. If we can show that

$$\sqrt[p]{\langle \|f(t+\epsilon) - f(t)\|^p \rangle} \leq C\epsilon^\alpha, \tag{A.3}$$

we have

$$\langle \|f(t+\epsilon) - f(t)\| \rangle \leq C\epsilon^\alpha. \tag{A.4}$$

Thus, we can estimate α by running a regression of

$$\frac{1}{p} \log \langle \|f(t+\epsilon) - f(t)\|^p \rangle \tag{A.5}$$

over $\log \epsilon$, where (25) corresponds to $p = 1$. We find from experiments that different p values give similar α estimates on 2-dimensional larval movement trajectories.

In (17), we used non-overlapping intervals to compute $\langle \|f(t+\epsilon) - f(t)\| \rangle$, reducing correlation between terms and enabling the application of the Chernoff bound in (24). Alternatively, overlapping intervals can be employed using a sliding window of length δ, defined as:

$$U(\delta) = \frac{1}{n-\delta} \sum_{i=1}^{n-\delta} \|\boldsymbol{x}_{i+\delta} - \boldsymbol{x}_i\|, \tag{A.6}$$

where the summands may exhibit correlation due to overlapping intervals. Despite this, we find that both (17) and (A.6) yield similar α estimates for larval movement data.

In (17), the computation begins at time 1. If we instead start from the o-th point, $U(\delta, o)$ can be computed as in (30). Then, $U(\delta)$ can be defined as the minimum over all starting offsets o, i.e., $U(\delta) = \min_{1 \le o < \delta} U(\delta, o)$. Similar to the argument in box-counting dimensions [5], taking the minimum over starting offsets does not affect the slope or the value of D_T as $\delta \to 1$.

Computing $U(\delta)$ requires a constant frame rate video to ensure that ϵ remains constant in (A.5). If the video has a variable frame rate, the average frame rate can be computed, and the coordinates of points at this average frame rate can be obtained from the original video using linear interpolation.

To examine the effect of p on D_T, we plot $\log_{10} U(\delta)$ vs. $\log_{10} \delta$ for the *Dysbindin* mutant movement trace in Fig. 2B with various values of p in (A.5) (Fig. S2). We compute $D_{T,0}$ using $\delta \in \{1, \ldots, 10\}$ and compare it with the long-range D_T using $\delta \in \{1, \ldots, 100\}$. The curves are nearly parallel across different p values when δ is small, indicating similar $D_{T,0}$. However, the long-range D_T increases with larger p, and $U(\delta)$ becomes slightly more irregular as p increases. Since D_T is best estimated at small δ, where p values yield similar results, $p = 1$ is the preferred choice for the temporal sampling algorithm in analyzing animal movement paths. We adopt $p = 1$ for the remainder of the study. This aligns with the recommendation in [40] that $p = 1$ is robust across all values of p, though their suggestion applies to one-dimensional time series.

Appendix A.2. Validation of D_T calculated using the temporal sampling algorithm

We validate the temporal sampling algorithm using examples of the Koch curve and Brownian motion, both of which have known FDs.

Koch Curve: Let $U(1) = 1$, meaning the distance between adjacent points on the lowest-level Koch curve is 1. At the next level, $U(2) = \sqrt{3}$. Using this, we calculate α as:

$$\alpha = \frac{\log U(2) - \log U(1)}{\log 2 - \log 1} = \frac{\log 3}{\log 4}. \tag{A.7}$$

The fractal dimension D_T is then:

$$D_T = \frac{1}{\alpha} = \frac{\log 4}{\log 3}, \tag{A.8}$$

which matches the true FD of the Koch curve [31].

Brownian Motion: For d-dimensional Brownian motion, $U(\delta) = \sqrt{d\delta\Delta t}$. This gives $\alpha = 1/2$, and the fractal dimension D_T becomes:

- $D_T = 3/2$ for $d = 1$,
- $D_T = 2$ for $d \geq 2$.

These results are consistent with [34, Theorem 4.29].

Validation with 1-Dimensional Time Series: For 1-dimensional time series ($d = 1$), we show that D_T in (21) aligns with well-established variation estimators. When $\alpha < 1$, we have:

$$D_T = 2 - \alpha. \tag{A.9}$$

This indicates the following:

- Using non-overlapping intervals as in (17) reproduces the Hall-Wood estimator.
- Using overlapping intervals as in (A.6) aligns with the variation estimators in [40].

However, the variation estimators in [40] apply only to 1-dimensional time series. In contrast, the proposed temporal sampling algorithm generalizes to time series of any dimensionality d, making it suitable for analyzing 2-dimensional animal movement paths.

Intuition Behind $D_T = 1/\alpha$: To understand why $D_T = 1/\alpha$, we observe that:

- $U(\delta)$ acts as the box size, and n/δ represents the number of boxes, analogous to box counting.
- D_B is the slope of $-\log(n/\delta)$ versus $\log U(\delta)$, which is equivalent to the slope of $\log \delta$ versus $\log U(\delta)$ with swapped axes.
- α, being the slope of $\log U(\delta)$ versus $\log \delta$, is the inverse of D_B.

Thus, $D_B \approx 1/\alpha = D_T$. While box counting provides finer control over box size ϵ, where ϵ can be any positive number, temporal sampling restricts δ to positive integers ($\delta \in \mathbb{N}$). To achieve finer resolution, the curve can be oversampled by a factor of a and then downsampled by a factor of b, resulting in an effective interval $\delta = a/b$.

Bounds on Fractal Dimensions D_T: It is important to note that D_T provides an upper bound on the Hausdorff dimension D_H, as stated in (21).

For many time series, including Brownian motion, this upper bound is exact [34]. Similarly to the box-counting dimension D_B, which serves as a common upper bound estimate for both the Hausdorff dimension D_H and D_B, we also employ the upper bound presented in (21) for quantifying FD. Generally, it holds that $D_H \leq D_T$.

By using D_T, we have the following Proposition.

Proposition 2: The FD D_T of any animal movement path has a dimension of 1 in the limit as the sampling time $\Delta t \to 0$.

To show this, we compute:

$$U(\Delta t) = \langle \|f(t+\epsilon) - f(t)\| \rangle = \frac{\sum_{i=1}^{n-1} \|f((i+1)\Delta t) - f(i\Delta t)\|}{T/\Delta t - 1}, \qquad \text{(A.10)}$$

where $n = T/\Delta t$. As $\Delta t \to 0$, we obtain:

$$\lim_{\Delta t \to 0} U(\Delta t) = \frac{\int_0^T \|f'(t)\| dt}{T} \Delta t = \bar{v}\Delta t, \qquad \text{(A.11)}$$

where $\bar{v} = \frac{\int_0^T \|f'(t)\| dt}{T}$ represents the average speed. The average speed of any animal is finite, constrained by physical limits such as the speed of light and the total kinetic energy available for movement. Consequently, as $\Delta t \to 0$, we have $\alpha = 1$ and $D_T = 1$ for any d. ■

There are several interesting aspects to consider regarding Proposition 2. Firstly, it is important to note that Proposition 2 holds only as $\Delta t \to 0$. For finite Δt, we might observe a local FD strictly greater than 1, emphasizing the scale-dependent nature of the FD. Secondly, this implies that with high-frequency sampling of the animal path, or equivalently, the use of a very high magnification magnifier, we can perceive a line locally at each point on the animal path where the animal is moving at a constant speed. Thirdly, Equation (A.11) suggests that the scale may be chosen proportionally to the average speed, assuming all animal paths are sampled at the same time interval Δt.

The total variation of Brownian motion is infinite [34], resulting in an infinite average speed—a trait unattainable by any physical animal. Although the FD of Brownian motion is 2 for $d \geq 2$, such a dimension cannot be achieved by any biological organism with non-zero mass at infinitesimal time intervals. This distinction highlights the fundamental differences between biological movement paths and purely stochastic processes like Brownian motion.

Appendix A.3. Correlation sum algorithm

The correlation sum is a widely used algorithm for computing the fractal dimension, specifically referred to as the correlation dimension in [3, Eq. (74)]. The correlation integral is defined as:

$$C(\epsilon) = \frac{1}{(n+1-w)(n-1)} \sum_{i=1}^{n} \sum_{\substack{j=1 \\ |i-j|\geq w}}^{n} I(\|\boldsymbol{x}_i - \boldsymbol{x}_j\| < \epsilon), \tag{A.12}$$

where $I(\cdot) = 1$ if its argument is true and 0 otherwise, and $w \geq 1$ is a correction introduced in [3] to eliminate spurious correlations arising from dense sampling of continuous dynamical systems. The definition of (A.12) can be expressed in words as:

$$C(\epsilon) = \frac{\begin{array}{c}\text{\# of distances less than } \epsilon \\ \text{excluding pairs of points closer together in time than } w\end{array}}{\text{\# of all distances}}. \tag{A.13}$$

When $w = 1$, (A.12) reduces to the original correlation integral [3, Eq. (44)].

The correlation dimension is defined as:

$$D_C = \lim_{\epsilon \to 0} \frac{\log C(\epsilon)}{\log \epsilon}. \tag{A.14}$$

Similar to (7), D_C can be estimated using a selected range of ϵ.

A maximum likelihood estimator is introduced in [36] to estimate the correlation dimension by incorporating all distances less than an upper bound ϵ_0. The Takens' estimator is given by:

$$D_{CT} = -\frac{m-1}{\sum\limits_{i=1}^{n} \sum\limits_{\substack{j=1, \\ |i-j|\geq w, \\ I(\|\boldsymbol{x}_i - \boldsymbol{x}_j\| < \epsilon_0)}}^{n} \log \frac{\|\boldsymbol{x}_i - \boldsymbol{x}_j\|}{\epsilon_0}}, \tag{A.15}$$

where

$$m = \sum_{i=1}^{n} \sum_{\substack{j=1, \\ |i-j|\geq w}}^{n} I(\|\boldsymbol{x}_i - \boldsymbol{x}_j\| < \epsilon_0) \tag{A.16}$$

is the total number of point pairs with distances less than ϵ_0. A commonly used choice for ϵ_0 is $\mathrm{std}(x)/4$, where $\mathrm{std}(x)$ denotes the standard deviation

of the data. For a fixed w, computing $C(\epsilon)$ in (A.13) has a time complexity of $O(n^2)$ and a space complexity of $O(1)$.

When comparing the correlation sum with box counting, we observe that the two methods operate in opposing manners. In box counting, smaller box sizes result in a larger number of boxes, leading to a decrease in $N(\epsilon)$ as ϵ increases. Conversely, in the correlation sum method, smaller distance thresholds result in fewer point pairs satisfying the distance condition, causing $C(\epsilon)$ to increase as ϵ grows. This distinction is evident from the fact that (3) has a minus sign while (A.14) does not. This difference suggests that the two estimators may be more effective in different scale regimes. For example, box counting may perform better at smaller scales, whereas the correlation sum might be better suited for larger scales.

From real larval movement data, we find that the correlation sum is highly sensitive to the parameter w, and the correlation dimension D_C varies significantly with the distance threshold ϵ. The choice of an appropriate distance for estimating the correlation dimension remains unclear. Moreover, the correlation sum algorithm produces dimension estimates that differ substantially from those obtained using other algorithms (Fig. S3). Not only does w greatly influence the $\log C(\epsilon)$ vs. $\log \epsilon$ curve, but the curve itself often fails to exhibit linearity (Fig. S3A). This observation aligns with findings in [41], which highlight the appearance of an anomalous shoulder in the correlation integral $C(\epsilon)$, potentially leading to inaccurate and spurious dimension estimates. To mitigate these issues, we incorporate corrections suggested in [41], using $w > 1$. While increasing w reduces the anomalous shoulder, the slope of the curve still exhibits considerable variation (Fig. S3B and S3C). Moreover, we observe that the slope changes dramatically with ϵ when using a rolling window of size 20. For instance, the fractal dimension of the *Dysbindin* mutant movement path differs markedly between $w = 1$ ($D_C = 1.21$) and $w = 30$ ($D_C = 1.78$, Fig. S3C). Although a larger w (e.g., $w = 30$) decreases slope variability, the magnitude of the slope still varies substantially from $w = 1$ to $w = 30$. This sensitivity to both w and ϵ may be an inherent limitation of the correlation sum algorithm when applied to real-world animal movement data.

Appendix A.4. Generalized fractal dimension

To compute $N(\epsilon)$ in the box-counting dimension, a box is counted if it contains at least one point. Intuitively, the number of points in each box can provide insights into the geometrical structure of the set. In [42, 43], the

generalized dimension was introduced by considering the number of points in the box.

Let $P_i(\epsilon)$ denote the probability of a randomly chosen point in the set X being located in the i-th box B_i. This can be estimated as the number of points in B_i divided by the total number of points n. Using the exact box-counting algorithm, we can compute the precise $P_i(\epsilon)$ for a piecewise linear curve by identifying the intersection points of the curve with the grid boundaries. The length of each line segment within a box is calculated, and its ratio to the total length of the curve gives $P_i(\epsilon)$. For boxes intersecting multiple line segments, the contributions from each segment are aggregated.

The generalized dimension is defined as:

$$D_q = \frac{1}{q-1} \lim_{\epsilon \to 0} \frac{\log \sum_i P_i^q(\epsilon)}{\log \epsilon} = \lim_{\epsilon \to 0} \frac{H_q(\epsilon)}{-\log \epsilon}, \tag{A.17}$$

where

$$H_q(\epsilon) = \frac{1}{1-q} \log \sum_i P_i^q(\epsilon). \tag{A.18}$$

When $q = 0$, D_q reduces to the original box-counting dimension in (3). When $q \to 1$, it becomes the information dimension introduced in [44]

$$D_I = \lim_{\epsilon \to 0} \frac{\sum_i P_i(\epsilon) \log P_i(\epsilon)}{-\log \epsilon}, \tag{A.19}$$

where the numerator is the Shannon's entropy.

We evaluate the $-\log_{10} H_q(\epsilon)$ versus $\log_{10} \epsilon$ plot for the *Dysbindin* mutant movement path in Fig. 2B across various q values, with $H_q(\epsilon)$ defined in (A.18). Interestingly, the curves for different q values are nearly parallel (Fig. S6A and S6B). To examine the sensitivity of D_q to origin shifts, we employ the same 20×20 origin shift grid used in Fig. 4, positioned at $3\epsilon^*$. While the average D_q values vary slightly, they remain notably consistent across different q values (Fig. S6C).

Additionally, D_q shows a monotonic increase for $q \leq 1.5$, consistent with the observation that $H_q(\epsilon)$ increases with q for a fixed ϵ. Consequently, $D_q(\epsilon)$ decreases with q as $\epsilon \to 0$. However, for finite scale ranges, this monotonicity may not hold; larger q values can lead to larger slopes and dimensions, particularly when $q > 1.5$. Notably, we observe that the coefficient of variation $\frac{\text{std}(D_q(\epsilon))}{\text{mean}(D_q(\epsilon))}$ increases as q increases (Fig. S6D).

Contrary to suggestions in [3], which advocate for better performance of generalized dimensions with $q > 1$, our findings suggest that $q = 0$, corresponding to the box-counting dimension, is preferable for analyzing animal movement paths, as it minimizes variation in D_q.

References

[1] J. Lauer, M. Zhou, S. Ye, W. Menegas, S. Schneider, T. Nath, M. M. Rahman, V. Di Santo, D. Soberanes, G. Feng, V. N. Murthy, G. Lauder, C. Dulac, M. W. Mathis, and A. Mathis. Multi-animal pose estimation, identification and tracking with deeplabcut. *Nat Methods*, 19(4):496–504, 2022.

[2] Felix Hausdorff. Dimension und äußeres maß. *Mathematische Annalen*, 79(1-2):157–179, March 1918.

[3] J. Theiler. Estimating fractal dimension. *Journal of The Optical Society of America A-optics Image Science and Vision*, 7(6):1055–1073, 1990.

[4] J. Aguirre, R.L. Viana, and M.A.F. Sanjuan. Fractal structures in nonlinear dynamics. *Rev. Mod. Phys.*, 81(1):333–386, 2009.

[5] J.Doyne Farmer, Edward Ott, and James A. Yorke. The dimension of chaotic attractors. *Physica D: Nonlinear Phenomena*, 7(1):153–180, 1983.

[6] B. Mandelbrot. How long is the coast of britain? statistical self-similarity and fractional dimension. *Science*, 155:636–638, 1967.

[7] Akhlaq Husain, Jaideep Reddy, Deepika Bisht, and Mohammad Sajid. Fractal dimension of coastline of australia. *Scientific Reports*, 11(1):6304, 2021.

[8] Hamid Sarkheil and Shahrokh Rahbari. Fractal geometry analysis of chemical structure of natural starch modification as a green biopolymeric product. *Arabian Journal of Chemistry*, 12(8):2430–2438, 2019.

[9] Yu-Chang Tzeng, Kuo-Tai Fan, and Kun-Shan Chen. A parallel differential box-counting algorithm applied to hyperspectral image classification. *IEEE Geoscience and Remote Sensing Letters*, 9(2):272–276, March 2012.

[10] Jiaxin Wu, Xin Jin, Shuo Mi, and Jinbo Tang. An effective method to compute the box-counting dimension based on the mathematical definition and intervals. *Results in Engineering*, 6:100106, 2020.

[11] Hamid Sarkheil, Shahrokh Rahbari, and Behzad Rayegani. Conversion based fuzzy fractal dimension integrating self-similarity and porosity, via dfs and fis (mamdani and sugeno systems). *Chaos, Solitons & Fractals*, 140:110183, 2020.

[12] Kimberly A. With. Using fractal analysis to assess how species perceive landscape structure. *Landscape Ecology*, 9(1):25–36, March 1994.

[13] T. O. Crist, D. S. Guertin, J. A. Wiens, and B. T. Milne. Animal movement in heterogeneous landscapes: An experiment with eleodes beetles in shortgrass prairie. *Functional Ecology*, 6(5):536, 1992.

[14] Kiho Im, Jong-Min Lee, Uicheul Yoon, Yong-Wook Shin, Soon Beom Hong, In Young Kim, Jun Soo Kwon, and Sun I. Kim. Fractal dimension in human cortical surface: Multiple regression analysis with cortical thickness, sulcal depth, and folding area. *Human Brain Mapping*, 27(12):994–1003, May 2006.

[15] J. M. Halley, S. Hartley, A. S. Kallimanis, W. E. Kunin, J. J. Lennon, and S. P. Sgardelis. Uses and abuses of fractal methodology in ecology. *Ecology Letters*, 7(3):254–271, February 2004.

[16] L. Y. Jan and Y. N. Jan. Properties of the larval neuromuscular junction in drosophila melanogaster. *J Physiol*, 262(1):189–214, 1976.

[17] H. Keshishian, K. Broadie, A. Chiba, and M. Bate. The drosophila neuromuscular junction: a model system for studying synaptic development and function. *Annu Rev Neurosci*, 19:545–75, 1996.

[18] G. W. Davis and M. Muller. Homeostatic control of presynaptic neurotransmitter release. *Annu Rev Physiol*, 77:251–70, 2015.

[19] C. A. Frank, M. J. Kennedy, C. P. Goold, K. W. Marek, and G. W. Davis. Mechanisms underlying the rapid induction and sustained expression of synaptic homeostasis. *Neuron*, 52(4):663–77, 2006.

[20] D.K. Dickman and G.W. Davis. The schizophrenia susceptibility gene dysbindin controls synaptic homeostasis. *Science*, 326(5956):1127–30, 2009.

[21] M. A. Benson, S. E. Newey, E. Martin-Rendon, R. Hawkes, and D. J. Blake. Dysbindin, a novel coiled-coil-containing protein that interacts with the dystrobrevins in muscle and brain. *J Biol Chem*, 276(26):24232–41, 2001.

[22] W. Li, Q. Zhang, N. Oiso, E. K. Novak, R. Gautam, E. P. O'Brien, C. L. Tinsley, D. J. Blake, R. A. Spritz, N. G. Copeland, N. A. Jenkins, D. Amato, B. A. Roe, M. Starcevic, E. C. Dell'Angelica, R. W. Elliott, V. Mishra, S. F. Kingsmore, R. E. Paylor, and R. T. Swank. Hermansky-pudlak syndrome type 7 (hps-7) results from mutant dysbindin, a member of the biogenesis of lysosome-related organelles complex 1 (bloc-1). *Nat Genet*, 35(1):84–9, 2003.

[23] C. A. Ghiani, M. Starcevic, I. A. Rodriguez-Fernandez, R. Nazarian, V. T. Cheli, L. N. Chan, J. S. Malvar, J. de Vellis, C. Sabatti, and E. C. Dell'Angelica. The dysbindin-containing complex (bloc-1) in brain: developmental regulation, interaction with snare proteins and role in neurite outgrowth. *Mol Psychiatry*, 15(2):115, 204–15, 2010.

[24] P. DeRosse, B. Funke, K. E. Burdick, T. Lencz, J. M. Ekholm, J. M. Kane, R. Kucherlapati, and A. K. Malhotra. Dysbindin genotype and negative symptoms in schizophrenia. *Am J Psychiatry*, 163(3):532–4, 2006.

[25] K. Talbot, W. L. Eidem, C. L. Tinsley, M. A. Benson, E. W. Thompson, R. J. Smith, C. G. Hahn, S. J. Siegel, J. Q. Trojanowski, R. E. Gur, D. J. Blake, and S. E. Arnold. Dysbindin-1 is reduced in intrinsic, glutamatergic terminals of the hippocampal formation in schizophrenia. *J Clin Invest*, 113(9):1353–63, 2004.

[26] A. Van Den Bogaert, J. Schumacher, T. G. Schulze, A. C. Otte, S. Ohlraun, S. Kovalenko, T. Becker, J. Freudenberg, E. G. Jonsson, M. Mattila-Evenden, G. C. Sedvall, P. M. Czerski, P. Kapelski, J. Hauser, W. Maier, M. Rietschel, P. Propping, M. M. Nothen, and

S. Cichon. The dtnbp1 (dysbindin) gene contributes to schizophrenia, depending on family history of the disease. *Am J Hum Genet*, 73(6):1438–43, 2003.

[27] R. E. Straub, Y. Jiang, C. J. MacLean, Y. Ma, B. T. Webb, M. V. Myakishev, C. Harris-Kerr, B. Wormley, H. Sadek, B. Kadambi, A. J. Cesare, A. Gibberman, X. Wang, F. A. O'Neill, D. Walsh, and K. S. Kendler. Genetic variation in the 6p22.3 gene dtnbp1, the human ortholog of the mouse dysbindin gene, is associated with schizophrenia. *Am J Hum Genet*, 71(2):337–48, 2002.

[28] D. Helmlinger and L. Tora. Sharing the saga. *Trends Biochem Sci*, 42(11):850–861, 2017.

[29] D. Qi, J. Larsson, and M. Mannervik. Drosophila ada2b is required for viability and normal histone h3 acetylation. *Mol Cell Biol*, 24(18):8080–9, 2004.

[30] T. Wang, D. T. Morency, N. Harris, and G. W. Davis. Epigenetic signaling in glia controls presynaptic homeostatic plasticity. *Neuron*, 105(3):491–505 e3, 2020.

[31] Kenneth Falconer. *Fractal Geometry: Mathematical Foundations and Applications, Second Edition*. Wiley, 2003.

[32] J. E. Bresenham. Algorithm for computer control of a digital plotter. *IBM Systems Journal*, 4(1):25–30, 1965.

[33] L.F. Richardson. The problem of contiguity: An appendix to statistics of deadly quarrels. *General System Yearbook*, 6:139–187, 1961.

[34] Peter Mörters and Yuval Peres. *Brownian Motion*. Cambridge University Press, Cambridge, 2010.

[35] S. A. Petersen, R. D. Fetter, J. N. Noordermeer, C. S. Goodman, and A. DiAntonio. Genetic analysis of glutamate receptors in drosophila reveals a retrograde signal regulating presynaptic transmitter release. *Neuron*, 19(6):1237–48, 1997.

[36] F. Takens. On the numerical determination of the dimension of an attractor. In Boele L. J. Braaksma, Hendrik W. Broer, and Floris Takens,

editors, *Dynamical Systems and Bifurcations*, pages 99–106, Berlin, Heidelberg, 1985. Springer Berlin Heidelberg.

[37] Ivan Markovsky and Sabine Van Huffel. Overview of total least-squares methods. *Signal Processing*, 87(10):2283–2302, 2007. Special Section: Total Least Squares and Errors-in-Variables Modeling.

[38] F. Geraci, R. Passiatore, N. Penzel, S. Laudani, A. Bertolino, G. Blasi, A. C. E. Graziano, G. C. Kikidis, C. Mazza, M. Parihar, A. Rampino, L. Sportelli, N. Trevisan, F. Drago, F. Papaleo, F. Sambataro, G. Pergola, and G. M. Leggio. Sex dimorphism controls dysbindin-related cognitive dysfunctions in mice and humans with the contribution of comt. *Mol Psychiatry*, 2024.

[39] Tilmann Gneiting and Martin Schlather. Stochastic models that separate fractal dimension and the hurst effect. *SIAM Review*, 46(2):269–282, January 2004.

[40] Tilmann Gneiting, Hana Ševčíková, and Donald B. Percival. Estimators of Fractal Dimension: Assessing the Roughness of Time Series and Spatial Data. *Statistical Science*, 27(2):247–277, 2012.

[41] James Theiler. Spurious dimension from correlation algorithms applied to limited time-series data. *Phys. Rev. A*, 34(3):2427–2432, September 1986.

[42] H. G. E. Hentschel and I. Procaccia. The infinite number of generalized dimensions of fractals and strange attractors. *Physica D: Nonlinear Phenomena*, 8(3):435–444, 1983.

[43] P. Grassberger. Generalized dimensions of strange attractors. *Phys. Lett. A*, 97(6):227–230, 1983.

[44] J. Balatoni and A. Rényi. Remarks on entropy. *Publications of the Mathematical Institute of the Hungarian Academy of Sciences*, 1:9–40, 1956.

FigureS1, Cui and Wang

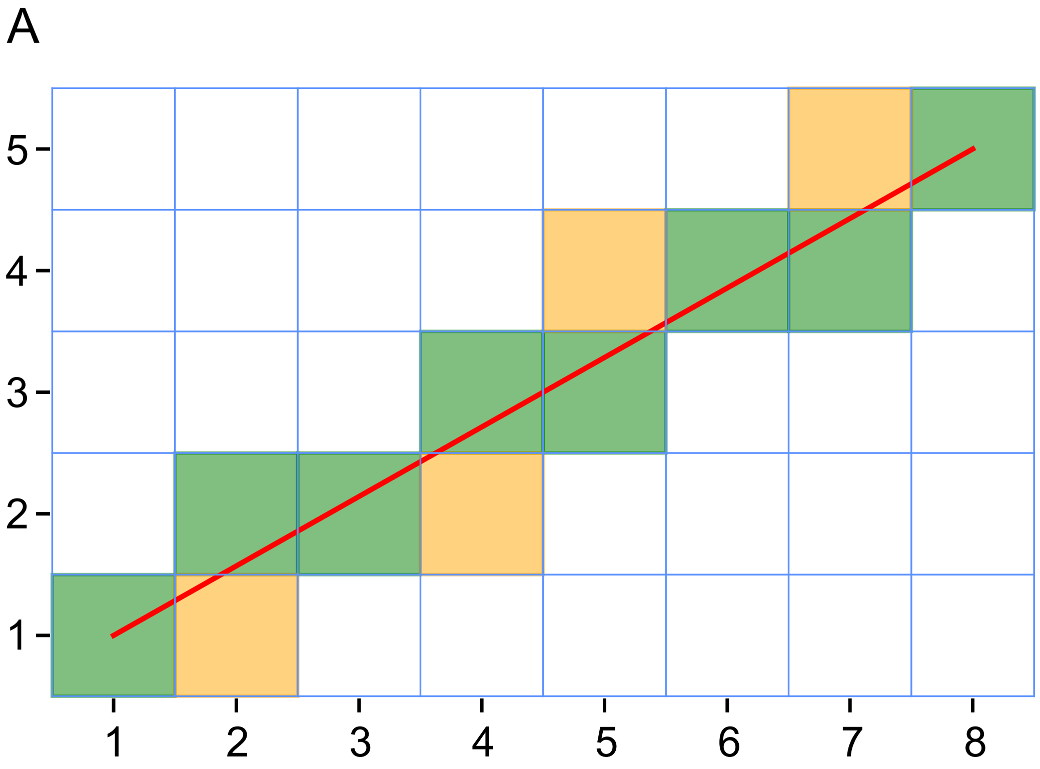

Figure S1: Bresenham's line algorithm does not identify all the boxes intersecting a line. **A.** An example of Bresenham's line algorithm is shown. Bresenham's line algorithm draws a line segment onto a 2-dimensional square grid by computing the coordinates of the pixels to form a close approximation to a straight line between two points. However, it does not always include all the pixels or "boxes" that intersect the line. Both the green and orange boxes intersect the red line, but only the green boxes are identified by Bresenham's line algorithm.

FigureS2, Cui and Wang

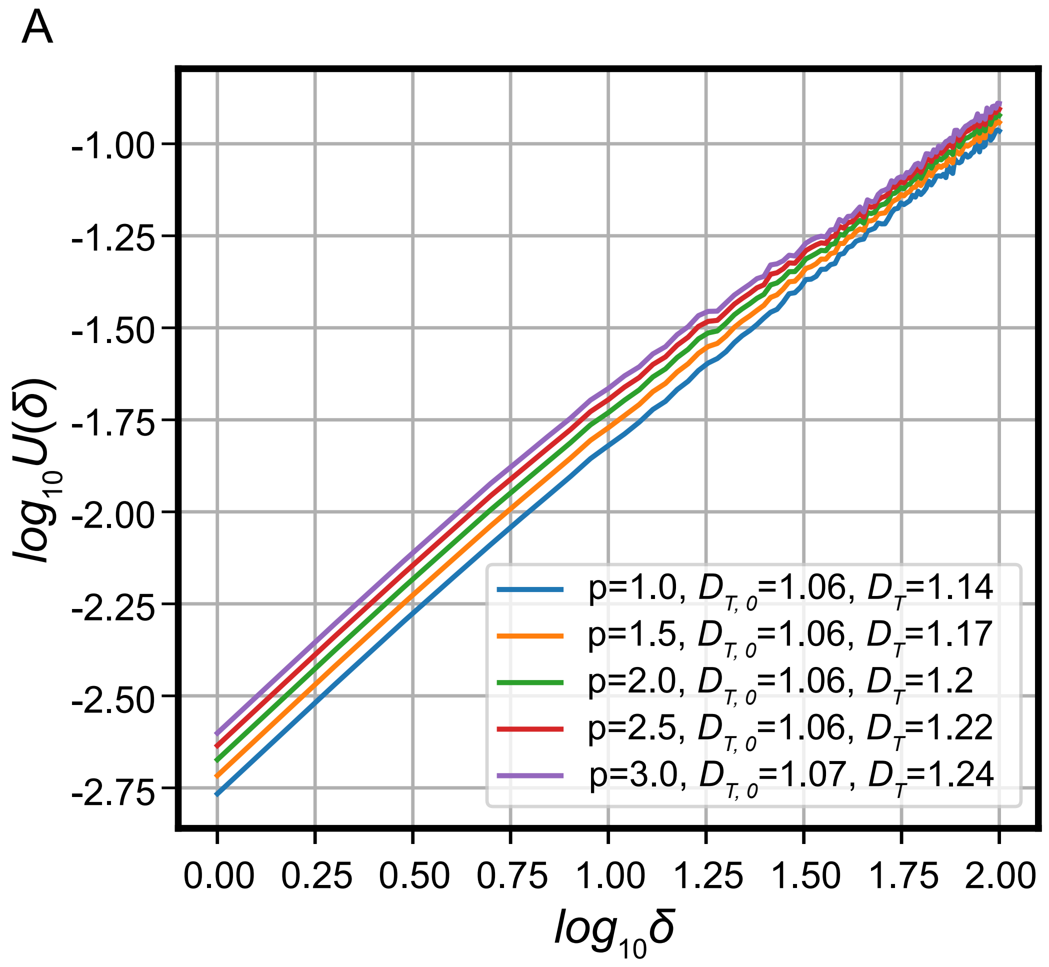

Figure S2: Variants of the temporal sampling algorithm. **A.** $\log_{10} U(\delta)$ vs $\log_{10}\delta$ plot for the *Dysbindin* mutant movement trace with different p in (A.5). The dimension $D_{T,0}$ calculated using $\delta \in \{1, \ldots, 10\}$ and D_T calculated using long-range $\delta \in \{1, \ldots, 100\}$ at different p are shown.

FigureS3, Cui and Wang

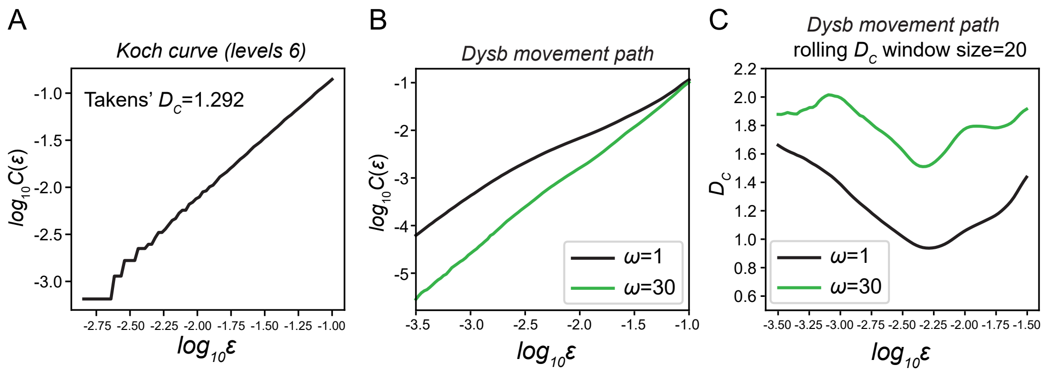

Figure S3: FD calculated using the correlation sum method. **A.** The $\log_{10} C(\epsilon)$ vs $\log\epsilon$ plot for the level 6 Koch curve calculated through the correlation sum method with the Takens' estimator ($D_C = 1.292$). **B.** The $\log_{10} C(\epsilon)$ vs $\log\epsilon$ plot for the movement path of the *Dysbindin* mutant calculated through the correlation sum method with $w = 1$ and $w = 30$. **C.** The local FD $D_C(\epsilon)$ estimated using a rolling window linear regression on the $\log_{10} C(\epsilon)$ vs $\log\epsilon$ with a window size of 20 for the *Dysbindin* mutant using the correlation sum method with $w = 1$ and $w = 30$.

FigureS4, Cui and Wang

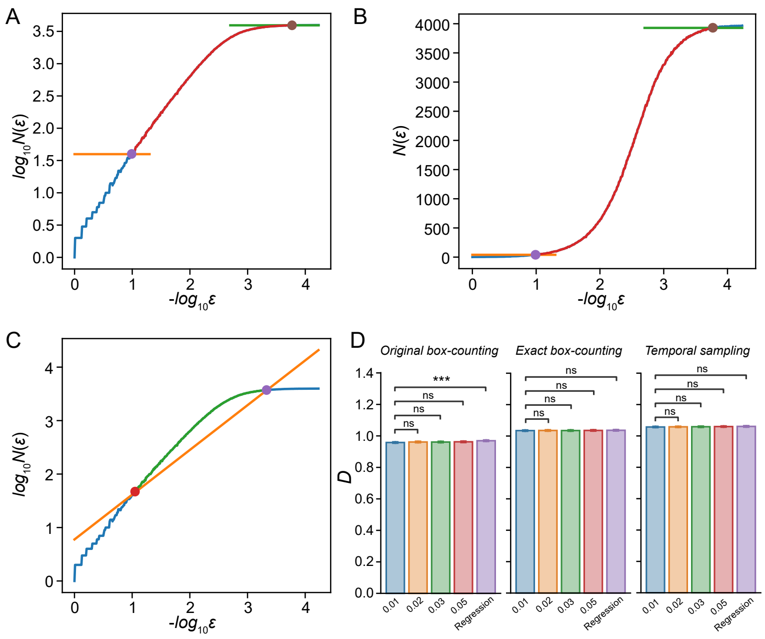

Figure S4: Impact of scale range on FD estimation. **A.** Example of applying Method 1 to the Dysbindin movement path shown in Figure 2B, using original box-counting with a threshold of $\gamma = 0.01$. The y-axis represents $\log_{10} N(\epsilon)$. The colored line between circles corresponds to the scale set $\mathcal{S}_1$ used for elbow point identification. **B.** As presented in A, but with the y-axis representing $N(\epsilon)$. The colored line between circles corresponds to the scale set $\mathcal{S}_1$ used for elbow point identification. **C.** Application of regression-based Method 2 to the *Dysbindin* movement path (shown in Figure 2B) using original box-counting . The colored line between circles corresponds to the scale set $\mathcal{S}_1$ used for elbow point identification. **D.** Comparison of FD estimates obtained using Method 1 with thresholds $\gamma = 0.01, 0.02, 0.03, 0.05$, and Method 2 across all 230 animal movement paths.

FigureS5, Cui and Wang

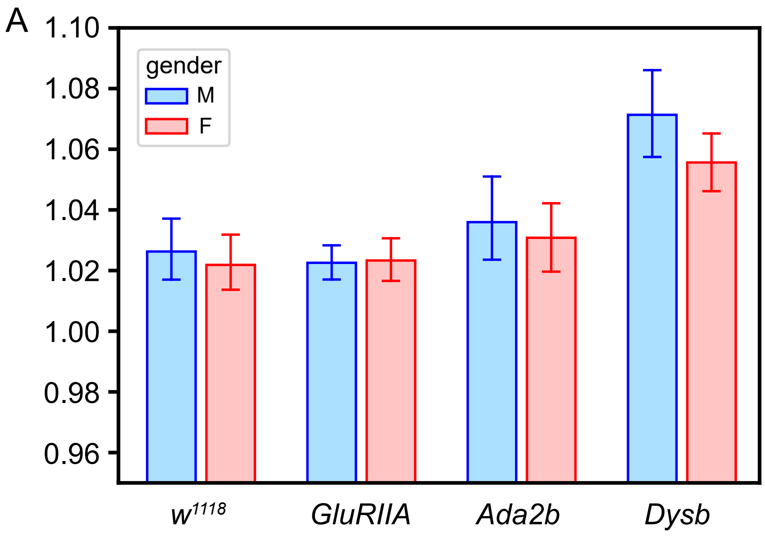

Figure S5: FD calculated for male and female animals using the original box-counting method. **A.** FD calculated for male and female animals of the *wild-type* (*w1118*, $n = 27$ male, 27 female) and mutant strains of *GluRIIA* ($n = 38, 38$), *Ada2b* ($n = 25, 25$), and *Dysbindin* (*Dysb*, $n = 25, 25$). *Mean* ± 95% confidence interval. *w1118* vs *Dysbindin* $p = 0.0937$, Mann-Whitney U test.

FigureS6, Cui and Wang

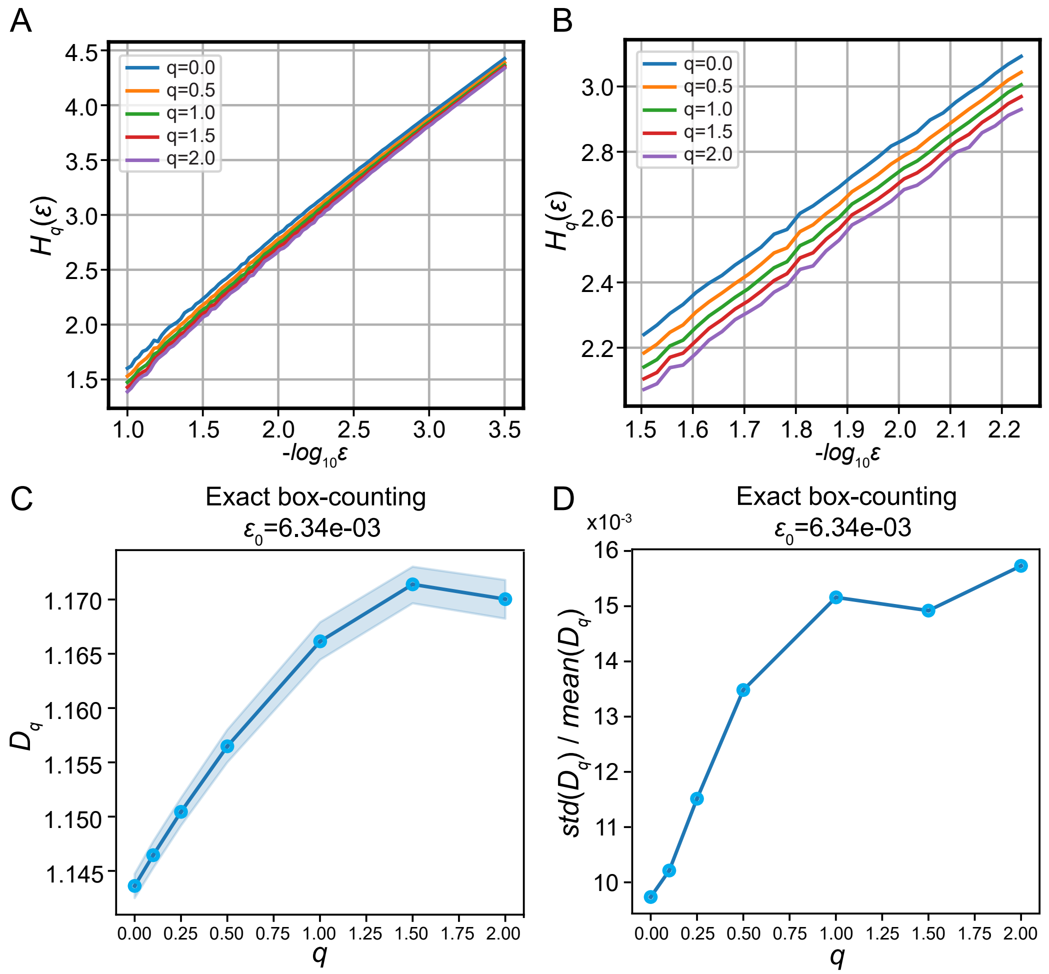

Figure S6: Generalized FD for the *Dysbindin* mutant. **A.** $\log_{10} H_q(\epsilon)$ vs $-\log_{10}\epsilon$ plot for the *Dysbindin* mutant movement path with different q, where $H_q(\epsilon)$ is defined in (A.18). **B.** $\log_{10} H_q(\epsilon)$ vs $-\log_{10}\epsilon$ plot for the *Dysbindin* mutant movement path with different q shown in higher magnification. **C.** D_q calculated using $3\epsilon^*$ with different q values for the *Dysbindin* mutant. **D.** The coefficient of variation of D_q ($\text{std}(D_q(\epsilon))/\text{mean}(D_q(\epsilon))$) increases with q for the *Dysbindin* mutant.
